# Gut stem cell aging is driven by mTORC1 via a p38 MAPK-p53 pathway

**DOI:** 10.1038/s41467-019-13911-x

**Published:** 2020-01-02

**Authors:** Dan He, Hongguang Wu, Jinnan Xiang, Xinsen Ruan, Peike Peng, Yuanyuan Ruan, Ye-Guang Chen, Yibin Wang, Qiang Yu, Hongbing Zhang, Samy L. Habib, Ronald A. De Pinho, Huijuan Liu, Baojie Li

**Affiliations:** 1 Bio-X Institutes, Key Laboratory for the Genetics of Developmental and Neuropsychiatric Disorders, Ministry of Education, Shanghai Jiao Tong University, Shanghai, 200240 China; 2 Department of Biochemistry and Molecular Biology, School of Basic Medical Sciences, Fudan University, Shanghai, 200032 China; 3 State Key Laboratory of Membrane Biology, Tsinghua-Peking Center for Life Sciences, School of Life Sciences, Tsinghua University, Beijing, 100084 China; 4 Department of Anesthesiology, Cardiovascular Research Laboratories, David Geffen School of Medicine, University of California, Los Angeles, CA 90095 USA; 5 A-STAR Genome Institute of Singapore, Singapore, 138648 Singapore; 6 State Key Laboratory of Medical Molecular Biology, Department of Physiology, Institute of Basic Medical Sciences, Chinese Academy of Medical Sciences and Peking Union Medical College, Beijing, 100730 China; 7 Department of Cellular and Structural Biology, University of Texas Health Science Center at San Antonio, San Antonio, TX78229 USA; 8 Department of Cancer Biology, University of Texas MD Anderson Cancer Center, Houston, TX 77030 USA; 9 Institute of Traditional Chinese Medicine and Stem Cell Research, School of Basic Medicine, Chengdu University of Traditional Chinese Medicine, Chengdu, 611137 China

**Keywords:** Cell biology, Ageing, Small intestine

## Abstract

Nutrients are absorbed solely by the intestinal villi. Aging of this organ causes malabsorption and associated illnesses, yet its aging mechanisms remain unclear. Here, we show that aging-caused intestinal villus structural and functional decline is regulated by mTORC1, a sensor of nutrients and growth factors, which is highly activated in intestinal stem and progenitor cells in geriatric mice. These aging phenotypes are recapitulated in intestinal stem cell-specific *Tsc1* knockout mice. Mechanistically, mTORC1 activation increases protein synthesis of MKK6 and augments activation of the p38 MAPK-p53 pathway, leading to decreases in the number and activity of intestinal stem cells as well as villus size and density. Targeting p38 MAPK or p53 prevents or rescues ISC and villus aging and nutrient absorption defects. These findings reveal that mTORC1 drives aging by augmenting a prominent stress response pathway in gut stem cells and identify p38 MAPK as an anti-aging target downstream of mTORC1.

## Abstract (web summary)

Intestinal aging is associated with declines in structure and absorption of nutrients. Here, the authors show that aging related intestinal decline is mediated by activation of the mTORC1-p38MAPK-p53 pathway in intestinal stem cells and can be ameliorated by abrogating mTORC1 or p38MAPK activity.

## Introduction

The incidence of aging-related disorders is increasing due to aging of the population^1^. Nutrient malabsorption is common among the elderly, and often causes anemia and other illnesses^2^. Nutrients are absorbed by the intestinal villi, which are composed of a layer of intestinal epithelial cells (IECs) and the lamina propria, and the absorption activity is affected by the size and density of villi^3^. The epithelial layer is renewed every 4–5 days by Lgr5^+^ intestinal stem cells (ISCs) following a symmetric division and neutral shift pattern^4–6^, which generate transient amplifying (TA) progenitor cells that later differentiate into absorptive or secretory cells^7^. The densities of crypts and villi are controlled by ISC fission^8^. In addition, Bmi1^+^ quiescent stem cells, TA cells, and secretory progenitors can revert to Lgr5^+^ ISCs after damage to regenerate the villi^9–13^. It has been reported that the number of ISCs and ISC regenerative activities are decreased in 17–24-month-old mice^14,15^, yet whether aging affects villus function and how villus aging is controlled remain less well understood.

mTOR, a sensor of nutrients and growth factors, is a central regulator of aging and a target for lifespan and healthspan extension^16–20^. mTOR forms mTORC1 and mTORC2 complexes, and mTORC1 activation promotes cell proliferation by increasing global protein synthesis and other anabolic processes^21–24^, which is assumed to cause irreversible cell senescence and/or oxidative and proteostatic stress and thus aging^25–28^. However, this hypothesis is not consistent with the reversible aging theory^29,30^. mTORC1 signaling has been shown to be required for IEC proliferation during homeostasis and regeneration^31–34^, including regeneration mediated by quiescent ISCs^35,36^. In addition, several studies have shown that diet restriction promotes Lgr5^+^ ISC expansion via mTORC1 signaling, although conflicting results have been reported regarding the exact roles played by mTORC1^14,37,38^. Nonetheless, the mechanisms by which mTORC1 signaling regulates ISCs and villus aging warrant further investigation.

The current genetic study reveals that mTORC1, which is hyperactivated in IECs, especially ISCs and TA cells of aged mice, drives villus aging by inhibiting ISC/progenitor cell proliferation through amplifying the MKK6-p38-p53 stress response pathway, with MKK6 protein synthesis being upregulated by mTORC1 activation. Villus aging can be slowed or prevented by targeting p38 MAPK or p53. These genetic results establish a prominent stress response pathway as an mTORC1 downstream effector in aging control, elucidate the mechanisms governing villus aging, and identify p38 MAPK as an antiaging target downstream of mTORC1 signaling.

## Results

### Aging causes a decrease in the numbers of crypts and TA cells

**Fig. 1:**
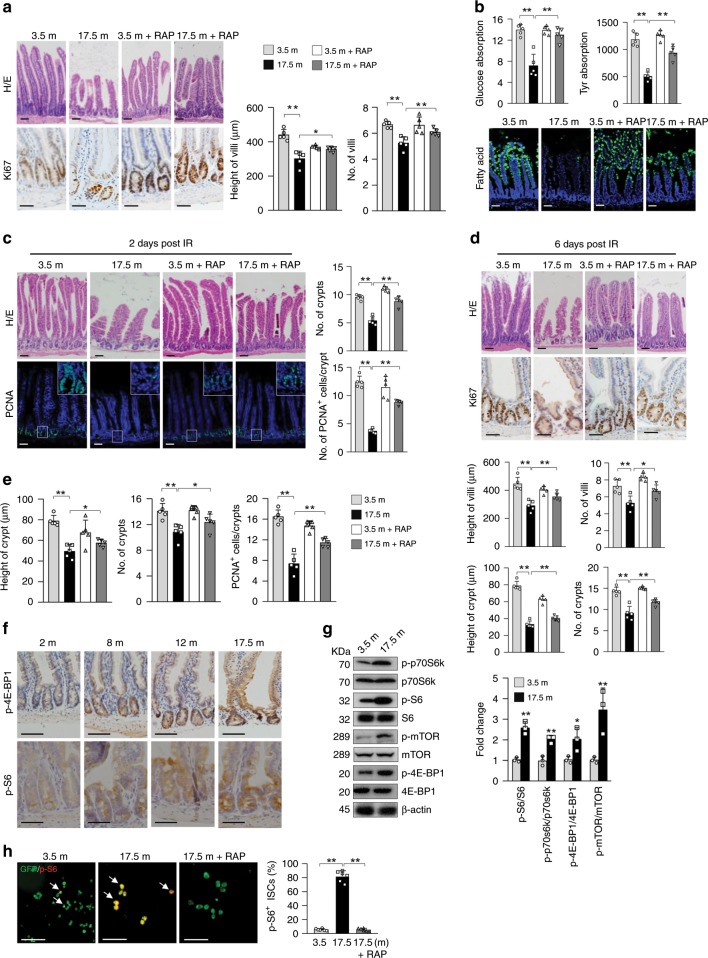
Enhanced mTORC1 activation in ISCs/TA cells of geriatric mice drives villus natural aging. **a**–**d** Seventeen and half-month-old mice showed deterioration in villus and crypt structures (right panels: quantitation data) (**a**), decreased nutrient absorption activities (**b**), increased sensitivity to IR-induced decreases in the numbers of crypts and proliferating cells at day 2 post IR (**c**), and compromised regeneration (decreases in the height and number of villi and crypts) at day 6 (**d**) compared with young mice, which were partially rescued by 1.5 months of RAP treatment (3 mg/kg body weight) starting at 16 months of age. Data are expressed as mean ± SEM. *N* = 5 mice per group. *P < 0.05, **P < 0.01 (determined using Student’s *t* test). **e** Seventeen and half-month-old mice showed decreases in the height and number of crypts and the number of proliferating TA cells (based on (a)), which were rescued by RAP. Data are expressed as mean ± SEM. *N* = 5 mice per group. *P < 0.05, **P < 0.01 (determined using Student’s *t* test). **f** Representative images (proximal jejunum midline sections) showed that mTORC1 activation was increased with age in crypt cells. **g** Western blot results showed that mTORC1 activation was increased in the crypt samples of 17.5-month-old mice compared with 3.5-month-old mice. Isolated crypts were directly lysed and used for WB analysis. Data are expressed as mean ± SEM. *N* = 3 mice per group. *P < 0.05, **P < 0.01 (determined using Student’s *t* test). **h** More Lgr5^+^ ISCs isolated from 17.5-month-old mice showed mTORC1 activation than those from 3.5-month-old mice, which was suppressed by RAP treatment. Lgr5^+^ ISCs were isolated from the small intestines of *Lgr5-GFP-CreERT* mice with FACS sorting and stained for p-S6. Right panel: quantification data (mean ± SEM). *N* = 6 mice per group. **P < 0.01 (determined using Student’s *t* test).

To understand aging of the nutrient-absorbing organ, we collected small intestines from normal B6 mice of various ages, which were bred and housed in the same facility. Histological analysis revealed that 16 or 17.5-month-old mice, compared with 2-, 3.5-, 8-, and 12-month-old mice, showed significant decreases in the height and number of villi in the proximal and distal jejunum (Fig. 1a; Supplementary Fig. 1a, b). The 17.5-month-old mice showed a 12% increase in body weight compared with 2–3-month-old mice. Mice at 24 months of age also showed villus structural deterioration (Supplementary Fig. 1c). These results indicate a decline in the absorption surface in aged mice, which are disagreeable with a recent study by Nalaparenddy et al. reporting an increase in the villus size in old mice^15^. While the causes of this discrepancy can be complex, one explanation could be that Nalaparenddy’s study used young and old mouse cohorts purchased from different suppliers^15^. We found that 16- or 17.5-month-old mice showed decreases in nutrient-absorbing activities for unmetabolizable L-glucose, amino acids, and fatty acids (Fig. 1b; Supplementary Fig. 1d).

The old mice also showed increased sensitivity to ionizing radiation (IR), manifested by greater decreases in the numbers of crypts and proliferating cells and greater increase in apoptotic cells than young mice at day 2 post IR (Fig. 1c; Supplementary Fig. 2a). This was associated with a decrease in PCNA and cyclin E and an increase in p53 in crypt samples (Supplementary Fig. 2b). Similar results were obtained at day 3 post IR (Supplementary Fig. 2c). The increased damage in old mice may be the cause of compromised villus regeneration observed at day 6 post IR, manifested by decreases in the height and number of villi and crypts (Fig. 1d; Supplementary Fig. 2d). Overall, these findings indicate that aging is associated with a deterioration of villus structure and function, increased sensitivity to stress, and compromised regeneration.

Old mice also showed decreases in the height and number of crypts, the ISC/progenitor-containing glands that control villus size and density. We observed a decrease in the number of Ki67^+^ progenitor cells (Fig. 1a, e; Supplementary Fig. 1a), but no significant changes in the numbers of apoptotic or senescent cells or differentiation of villus cells after normalized to the villus size (Supplementary Fig. 2e–g). Although the numbers of villi and crypts were decreased in old mice, the crypt-to-villus ratio was unaltered (Supplementary Fig. 2h), suggesting that aging-related decreases in villus height and density may be caused by decreases in the numbers of proliferating TA cells and crypts, respectively. Yilmaz’ group also reported that aging caused decreases in the number and regeneration ability of ISCs, but not defect in goblet or enterocyte differentiation^14^. Taken together, our and others’ studies suggest that villus aging-associated decrease in villus size and density are likely caused by defects in ISCs and TA progenitors^14,15^.

### Hyperactivated mTOR in IECs contributes to villus aging

mTOR activation is implicated in the aging process^34,39^. Immunostaining showed that p-4E-BP1 and p-S6, indicators of mTORC1 activation, were increased with age in IECs, especially in crypt cells (Fig. 1f). Western blot analysis confirmed an increase in mTORC1 activation in crypt samples of old mice (Fig. 1g). Because the number of ISCs was too limited for western blot analysis, we sorted Lgr5^+^ ISCs from 3.5- and 17.5-month-old *Lgr5-GFP-CreERT* mice and immunostained them for p-S6. We found that substantially more ISCs displayed mTORC1 activation in old mice (Fig. 1h). The factors that cause mTORC1 hyperactivation in aged IECs and ISCs may include systemic and niche cues and, warrant further investigation^39,40^.

Interestingly, treatment of 16-month-old mice with rapamycin (RAP), an mTORC1 inhibitor^21^, for 1.5 months inhibited mTORC1 activity (Supplementary Fig. 2i) and partially rescued aging-like phenotypes, including decreases in villus height and density and nutrient absorption activities, increased sensitivity to IR at day 2, and compromised villus regeneration at day 6 post IR (Fig. 1a–d; Supplementary Fig. 2a–d). It has been previously reported that acute RAP inhibited villus regeneration in normal mice^31,33,36^. Here, we carried out the above experiments 3 days after completion of drug treatment to avoid possible acute effects of RAP. Moreover, RAP mainly rescued IR-induced decrease in cell proliferation and increase in apoptosis at day 2 or 3 post IR (Fig. 1c; Supplementary Fig. 2a–c), which might help villus regeneration. In addition, RAP partially rescued the decreases in the height and number of crypts and the number of proliferating TA cells in old mice (Fig. 1a, e). These results suggest that enhanced mTORC1 activation contributes to villus natural aging.

### *Tsc1* ablation in IECs causes villus premature aging

To test whether mTORC1 activation causes villus aging, we ablated *Tsc1*, a disease gene encoding an mTOR suppressor, using *Villin-Cre* mice. Villin is expressed in all small intestinal IECs, including ISCs, but not colon crypts^41^. *Villin-Cre;Tsc1*^*f/f*^ mice exhibited enhanced mTOR activation in the whole villi (Supplementary Fig. 3a). We found that 2- or 3-month-old *Villin-Cre;Tsc1*^*f/f*^ mice showed increases in the number and size of crypts, but insignificant increases in villus size (Supplementary Fig. 3b). The nutrient absorption rates were not altered in these mice (Supplementary Fig. 3c), suggesting that mTORC1 activation in IECs per se does not affect nutrient absorption in the presence of normal villi. Surprisingly, 4–6-month-old mutant mice showed villus/crypts similar to age-matched wild-type mice (Supplementary Fig. 3b).

Starting from 7 months of age, the mutant mice displayed aging-related changes, including decreases in villus height and density, nutrient absorption activities, and villus regeneration (Supplementary Fig. 3b–d). The compromised regeneration in *Villin-Cre;Tsc1*^*f/f*^ mice was likely caused by increases in IR-induced cell death and villus damage at day 2 post IR (Supplementary Fig. 3d). Overall, these results indicate that *Tsc1* deletion in IECs cause villus premature aging.

### *Tsc1* ablation in ISCs-induced villus aging is rescued by RAP

The above studies show that nutrient absorption defects observed in villus natural or premature aging models are associated with decreases in villus size and density, whereas mTORC1 activation per se in IECs does not affect nutrient absorption activities at young age (Supplementary Fig. 3c). Since villus size and density are controlled by ISC and progenitor cells, we ablated *Tsc1* in ISCs using *Lgr5-CreERT* mice^4^. *Lgr5-CreERT* reportedly only labels part of ISCs after tamoxifen (TAM) administration and generates chimeric labeling patterns^4^. Our lineage-tracing studies revealed that three doses of TAM administered daily to 1-month-old *Lgr5-CreERT;tdTomato* mice labeled 65% of the crypts by 2 months of age (Supplementary Fig. 4a). A majority of the cells in almost all the villi were Tomato-labeled, likely because each villus contains at least six crypts^7^.

**Fig. 2:**
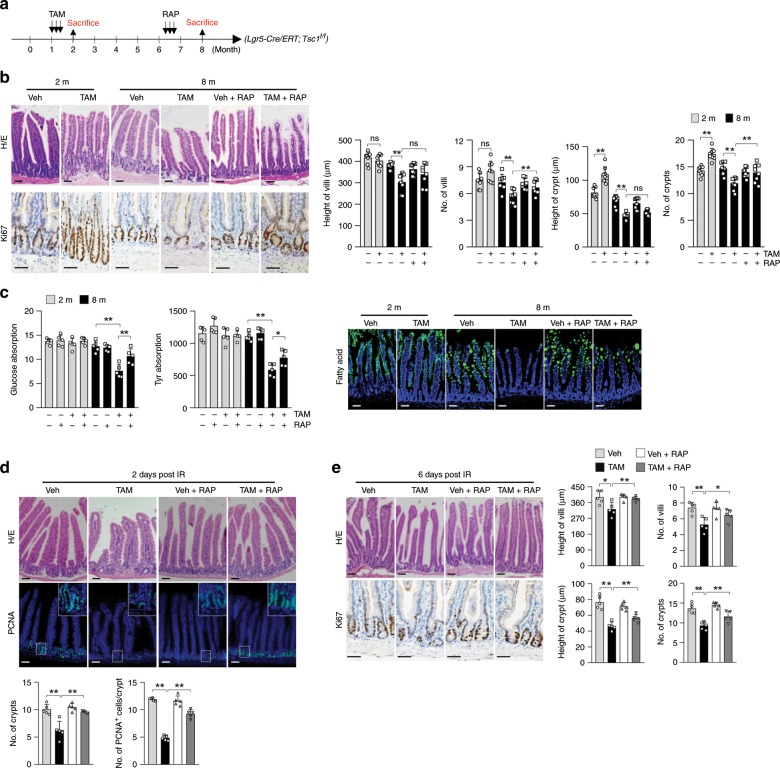
Deletion of *Tsc1* in Lgr5^+^ ISCs leads to villus premature aging. **a** A schematic for conditional ablation of *Tsc1* and RAP treatment in *Lgr5-CreERT;Tsc1*^*f/f*^ mice (**b**–**e**). **b** H/E and Ki67 staining showed that ablation of *Tsc1* in Lgr5^+^ ISCs led to crypt overgrowth at 2 months of age and deterioration of villus structure (the midline section of the proximal jejunum) at 8 months of age, which were partially rescued by RAP. Right panels: quantification data (mean ± SEM). *N* = 8 mice per group. **P < 0.01 (determined using Student’s *t* test). **c** Eight-month-old *Lgr5-CreERT;Tsc1*^*f/f*^ mice showed decreased nutrient absorption activities for L-glucose (mmol/l), amino acids (ng/ml), and fatty acids, which were partially rescued by RAP treatment. Data are expressed as mean ± SEM. *N* = 5 mice per group. *P < 0.05, **P < 0.01 (determined using Student’s *t* test). **d**, **e** Eight-month-old *Lgr5-CreERT;Tsc1*^*f/f*^ mice showed increased sensitivity to IR-induced decreases in the numbers of crypts and proliferating cells at day 2 post IR (**d**), and compromised regeneration (decreases in the height and number of villi and crypts) at day 6 post IR (**e**), which were partially rescued by RAP treatment. Data are expressed as mean ± SEM. *N* = 5 mice per group. *P < 0.05, **P < 0.01 (determined using Student’s *t* test).

We found that TAM-induced *Tsc1* ablation in *Lgr5-CreERT;Tsc1*^*f/f*^ mice at 1 month of age led to an increase in mTORC1 activation in most of the cells of almost all the villi by 2 months of age (Fig. 2a; Supplementary Fig. 4b). These mice showed increases in the size and number of crypts, but insignificant increases in villus size (Fig. 2b). The nutrient absorption activities were unaltered in these mutant mice (Fig. 2c), confirming that mTORC1 activation in IECs per se does not affect their nutrient absorption activities. However, mice of 7 months of age or older displayed a slight but insignificant decrease in body weight and premature aging phenotypes, including changes in villus height and density (proximal and distal jejunum) and nutrient absorption activities (Fig. 2b, c; Supplementary Fig. 5a).

The mutant mice also showed increased sensitivity to IR, manifested by greater decreases in the numbers of crypts and proliferating cells and greater increase in apoptotic cells than control mice at day 2 post IR (Fig. 2d; Supplementary Fig. 5b), associated with a decrease in PCNA and cyclin E and an increase in p53 in crypt samples (Supplementary Fig. 5c). Similar results were obtained at day 3 post IR (Supplementary Fig 5d). This might have led to compromised villus regeneration at day 6 post IR (Fig. 2e). Although mTOR signaling is required for villus regeneration^31,33,36^, we found that mTOR hyperactivation sensitized the cells to IR-induced damage, which might delay the regeneration process. Note that most of the crypts and almost all the villi showed increased mTORC1 activation in 8-month-old *Lgr5-CreERT; Tsc1*^*f/f*^ mice to a greater extent than that in 2-month-old *Lgr5-CreERT; Tsc1*^*f/f*^ mice (Supplementary Fig. 4b).

The villus premature aging phenotypes of *Lgr5-CreERT; Tsc1*^*f/f*^ mice could be partially rescued by 1.5 months of RAP treatment starting at 6.5 months of age (Fig. 2a–e). This included the rescue of IR-induced decrease in cell proliferation and increase in apoptosis at day 2 or 3 post IR (Fig. 2d; Supplementary Fig. 5d). RAP could also inhibit mTORC1-driven crypt overgrowth in *Lgr5-CreERT;Tsc1*^*f/f*^ mice at 2 months of age (Supplementary Fig. 5e), suggesting that villus growth and aging are at least partially mediated by elevated mTORC1 activation. In addition, *Tsc1* deletion led to an overgrowth phenotype in colon crypts at 2 months of age, followed by crypt atrophy at 7 months of age, which were rescued by RAP (Supplementary Fig. 5b). These results revealed biphasic effects of mTORC1 activation on Lgr5^+^ ISCs in the small intestine and colon.

Our findings that (i) mTORC1 is greatly activated in ISC and progenitor cells of aged mice; (ii) nutrient absorption is normal even when mTORC1 is activated in IECs of young *Lgr5-CreERT;Tsc1*^*f/f*^ mice and *Villin-Cre;Tsc1*^*f/f*^ mice, which have normal villus size and density; (iii) aging-related nutrient absorption decline is always associated with decreases in villus size and density, which are controlled by Lgr5^+^ ISCs, suggesting that activated mTORC1 promotes villus aging mainly through ISCs.

### mTORC1-driven villus aging may not rely on overgrowth

Both *Villin-Cre;Tsc1*^*f/f*^ mice and *Lgr5-CreERT;Tsc1*^*f/f*^ mice showed increases in the size of crypts at 2–3 months of age before showing villus premature aging at 7–8 months of age (Fig. 2b; Supplementary Fig. 3b). Is it possible that villus overgrowth at 2–3 months of age causes villus premature aging later? Our observation that RAP administration to 6.5-month-old mice could rescue villus aging phenotypes, suggests that villus premature aging is not an inevitable consequence of mTORC1-driven crypt overgrowth at 2–3 months of age (Fig. 2b–e); such rescue may not occur if mTORC1-driven aging is caused by irreversible ISC/TA cell replicative senescence and/or apoptosis. For validation, we induced *Tsc1* ablation in *Lgr5-CreERT;Tsc1*^*f/f*^ mice at 6.5 months of age. These mice displayed aging-like phenotypes at 8 months of age, including changes in villus height and density and nutrient absorption activities (Supplementary Fig. 6a–c). However, TAM administration failed to induce crypt overgrowth at 7.5 months of age (Supplementary Fig. 6d). Overall, these results suggest that mTORC1-driven villus premature aging may not rely on mTORC1-driven crypt overgrowth.

### mTORC1 activation eventually suppresses ISC proliferation

**Fig. 3:**
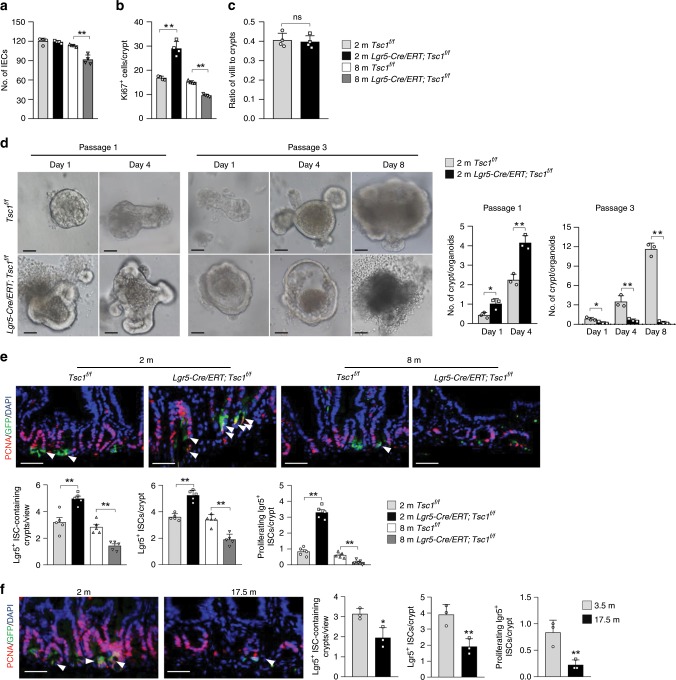
*Tsc1* deletion leads to defects in propagation of organoids and ISC/progenitor cells. **a**, **b** Eight-month-old *Lgr5-GFP-CreERT;Tsc1*^*f/f*^ mice showed decreases in the number of IECs (**a**) and the number of proliferating TA cells (**b**) (based on Fig. 2b). Data are expressed as mean ± SEM. *N* = 4 mice per group. **P < 0.01 (determined using Student’s *t* test). **c** Eight-month-old *Lgr5-GFP-CreERT;Tsc1*^*f/f*^ mice showed no change in the villus-to-crypt ratios. *N* = 4. **d** Representative images showing that *Tsc1*^*−/−*^ crypts (isolated from *Villin-Cre;Tsc1*^*f/f*^ mice, since *Lgr5-GFP-CreERT* can label ~65% of the crypts) displayed increases in the size of the minigut organoids and the number of crypts, whereas serial passaging of the organoids revealed that *Tsc1*-deficient organoids quickly lost their ability to propagate. Right panels: quantification of the data (mean ± SEM). *N* = 3 mice per group. *P < 0.05, **P < 0.01 (determined using Student’s *t* test). **e** Eight-month-old *Lgr5-GFP-CreERT;Tsc1*^*f/f*^ mice showed decreases in the numbers of ISCs and proliferating ISCs. Intestine sections were immunostained for PCNA, and cells positive for PCNA and GFP were counted and normalized to the total numbers of Lgr5^+^ ISCs. Bottom panels: quantitation data (mean ± SEM). *N* = 5 mice per group. **P < 0.01 (determined using Student’s *t* test). **f** The numbers of ISCs and proliferating ISCs were decreased in naturally aged 17.5-month-old mice. Right panels: quantitation data (mean ± SEM). *N* = 3 mice per group. *P < 0.05, **P < 0.01 (determined using Student’s *t* test).

What caused the decreases in villus height and density in 8-month-old *Lgr5-CreERT;Tsc1*^*f/f*^ mice? We found that the decrease in villus height was accompanied by decreases in the number of IECs and the number of Ki67^+^ progenitors (Figs. 2b, 3a, b). On the other hand, IEC apoptosis, senescence, or differentiation were not significantly altered (Supplementary Fig. 5f–h). In addition, the decrease in the number of villi was accompanied by a decrease in the number of crypts (Fig. 2b). Since the villus-to-crypt ratio was not altered (Fig. 3c), we conclude that decreased villus density is caused by decreased numbers of crypts. Overall, these results suggest that the decreases in villus size and density in *Lgr5-CreERT;Tsc1*^*f/f*^ mice at 7–8 months of age are caused by defects in the crypts and TA cell proliferation.

We then used ex vivo organoid cultures to verify these findings. We isolated crypts from 2-month-old normal or *Villin-Cre; Tsc1*^*f/f*^ mice and performed ex vivo minigut organoid cultures. *Tsc1*-deficient crypts showed increased growth in organoid cultures; however, they lost the ability to propagate at passage 3 (Fig. 3d), confirming the biphasic roles of mTORC1 signaling in crypt growth and aging.

*Tsc1* ablation-induced premature villus aging was also associated with defects in ISCs. At 8 months of age, the number of crypts containing Lgr5-expressing ISCs and the number of Lgr5^+^ ISCs per crypt were decreased, whereas the opposite results were observed at 2-month-old *Lgr5-CreERT;Tsc1*^*f/f*^ mice (Fig. 3e). Moreover, the number of proliferating Lgr5^+^ ISCs was also decreased in 8-month-old *Lgr5-CreERT;Tsc1*^*f/f*^ mice (Fig. 3e). Similarly, the numbers of Lgr5^+^ ISCs and proliferating Lgr5^+^ ISCs were decreased in naturally aged mice (Fig. 3f). Overall, these results suggest that both *Tsc1* deficiency-induced villus premature aging and natural aging are likely caused by decreases in the number of ISC/TA cells.

### mTORC1 increases MKK6 protein synthesis and p38 activation

**Fig. 4:**
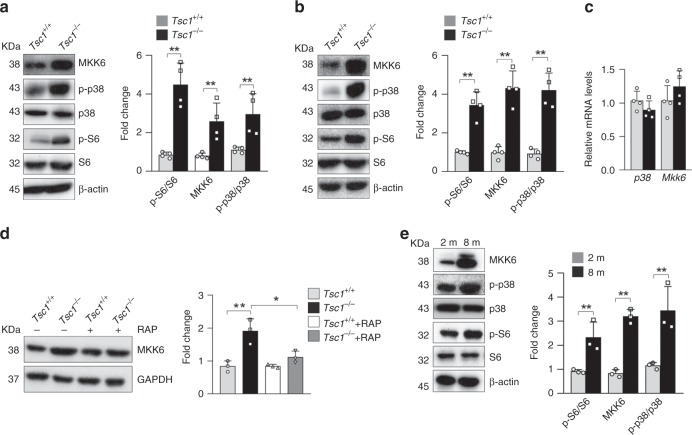
*Tsc1* deficiency increases MKK6 protein levels in an mTORC1-dependent manner. **a**, **b** Western blot results showed that the levels of MKK6 and activation of p38 MAPKs were increased in villus samples of *Villin-Cre; Tsc1*^*f/f*^ mice (**a**) and *Tsc1*-deficient organoids (**b**) compared with those in controls. Right panels: quantification data (mean ± SEM). *N* = 4 mice for **a** and 3 for **b**. **P < 0.01 (determined using Student’s *t* test). **c** *Tsc1* deficiency did not significantly affect the mRNA levels of *Mkk6* in crypt samples. The total RNA was isolated from the crypt samples, converted into cDNA, and then used for quantitative PCR analysis. *N* = 4 mice per group. **d** *Tsc1* deficiency led to an increase in radio-labeled methionine incorporation into MKK6, which were suppressed by RAP. Primary enteroblast cultures were pulse-labeled with ^35^S Met and Cys for 1 h. The cells were collected, and MKK6 was immunoprecipitated and fractionated onto SDS-PAGE gels, which were dried; the signals were detected with a PhosphorImager. Right panels: quantification data (mean ± SEM). *N* = 3 mice per group. *P < 0.05 (determined using Student’s *t* test). **e** Western blot analysis revealed that crypt samples from 8-month-old *Lgr5-GFP-CreERT;Tsc1*^*f/f*^ mice showed increases in MKK6 expression, p38 MAPK activation, and mTORC1 activation compared with those from 2-month-old mice. Right panels: quantification data (mean ± SEM). *N* = 3 per group. *P < 0.05 (determined using Student’s *t* test).

To understand the molecular basis underlying mTORC1-driven villus premature aging and decreased ISC/TA cell proliferation, we used antibody arrays (204 antibodies) to compare the expression and activation of proteins involved in cell proliferation. To this end, we compared snap-frozen intestine samples of *Villin-Cre;Tsc1*^*f/f*^ and control mice to minimize possible effects of crypt isolation procedures on protein phosphorylation. We found that *Tsc1* deficiency altered the expression and activation of several signaling molecules, including p38 MAPK, MEK1, and p53 (Supplementary Table 1). We chose p38 MAPKs for further studies since they are stress-responsive kinases that are involved in aging^42^. Western blot analysis revealed that the levels of MKK6 protein and p38 MAPK activation were increased in *Tsc1*^*−/−*^ villus samples and *Tsc1*^*−/−*^ organoid samples (Fig. 4a, b). Mechanistically, *Tsc1* deficiency did not significantly affect the mRNA levels of *Mkk6* in crypt samples (Fig. 4c). Instead, incorporation of radio-labeled methionine into MKK6 was increased in *Tsc1*^*−/−*^ primary enteroblasts, which were suppressed by RAP treatment (Fig. 4d). Increases in MKK6 protein and p38 MAPK activation were also observed in *Tsc1*^*−/−*^ MEFs (Supplementary Fig. 7a–c). We found that transient knockdown of MKK6 with siRNA led to a decrease in p38 MAPK activation (Supplementary Fig. 7d), indicating that elevated MKK6 is responsible for enhanced p38 MAPK activation in *Tsc1*-deficient cells. mTORC1 has been shown to increase protein synthesis of transcripts with 5′ terminal oligopyrimidine (TOP) or TOP-like motifs^43^. Mouse *Mkk6* transcripts contain several TOP or TOP-like tracts in the 5′ untranslated region (Supplementary Fig. 7e). These results suggest that mTORC1 activation promotes protein synthesis of MKK6 and thus enhances p38 MAPK activation.

Moreover, comparison of crypt samples of 2- and 8-month-old *Lgr5-CreERT;Tsc1*^*f/f*^ mice by western blot revealed that p38 MAPK activation was further augmented in the old mice, accompanied by an increase in mTORC1 activation (Fig. 4e). Immunostaining results confirmed enhanced p38 activation in the crypts of old mice (Supplementary Fig. 7f). Increased mTORC1 activation was also observed on intestine sections of 8-month-old mice compared with 2-month-old mice (Fig. 1f; Supplementary Fig. 4b). These results suggest that extrinsic and/or intrinsic changes at 7–8 months of age may be involved in enhanced activation of mTORC1 and p38 MAPKs in ISCs and IECs.

### *Mapk14* ablation prevents mTORC1-driven villus and ISC aging

Because the p38 MAPK pathway plays a negative role in cell proliferation under most conditions^42^, enhanced p38 MAPK activation may underlie mTORC1-driven ISC/progenitor aging. To test this possibility, we ablated *Mapk14*, which encodes p38α, in *Villin-Cre;Tsc1*^*f/f*^ mice to circumvent the possible complication of chimeric ablation of *Tsc1* and *Mapk14*. We found that 2-month-old *Villin-Cre;Mapk14*^*f/f*^ mice, like *Villin-Cre;Tsc1*^*f/f*^ mice, showed decreased p38α expression and increases in the number and size of crypts (Supplementary Fig. 8a, b), as previously reported^44^. Moreover, *Mapk14* ablation prevented the development of aging-related histological phenotypes, leading to crypt overgrowth and fusion in 8-month-old *Villin-Cre;Tsc1*^*f/f*^ mice (Supplementary Fig. 8b).

**Fig. 5:**
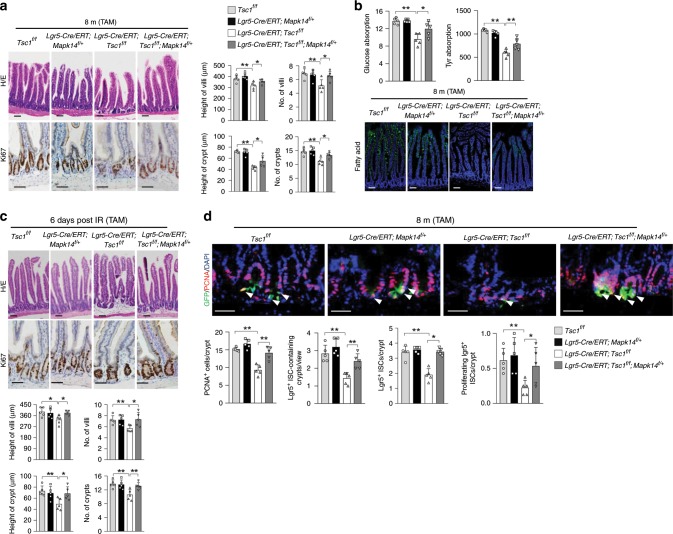
MKK6-p38α MAPK signaling mediates villus premature aging caused by *Tsc1* deficiency. **a**–**c** *Mapk14* haplodeficiency rescued the deterioration in villus structures (**a**), decreased nutrient absorption activities (**b**), and compromised regeneration (**c**) at 8 month of age. Data are expressed as mean ± SEM. *N* = 5 mice per group. *P < 0.05, **P < 0.01 (determined using Student’s *t* test). **d** *Mapk14* haplodeficiency rescued the decreases in the numbers of ISCs and proliferating ISCs in 8-month-old *Lgr5-GFP-CreERT;Tsc1*^*f/f*^ mice. The intestinal sections were immunostained for PCNA, and cells positive for PCNA and GFP were counted and normalized to the number of Lgr5^+^ ISCs. Bottom panels: quantitation data (mean ± SEM). *N* = 5 mice per group. *P < 0.05, **P < 0.01 (determined using Student’s *t* test).

We also ablated *Mapk14* in *Lgr5-CreERT;Tsc1*^*f/f*^ mice (Supplementary Fig. 9a), and found that deletion of just one allele of *Mapk14* prevented the development of villus premature aging phenotypes at 8 months of age, including defects in villus structure, nutrient absorption activities, and villus regeneration at day 6 post IR (Fig. 5a–c). *Mapk14* haploinsufficiency also rescued increased sensitivity to IR at day 2 post IR (Supplementary Fig. 9b–d), the decrease in the numbers of proliferating TA cells, Lgr5^+^ ISC-containing crypts, Lgr5^+^ ISCs per crypt, and proliferating Lgr5^+^ ISCs, as well as mTORC1-driven colon crypt atrophy (Fig. 5d; Supplementary Fig. 9e). Overall, these genetic data suggest that p38 MAPKs mediate mTORC1-driven ISC and villus premature aging. A recent study reported that mitochondria-produced reactive oxygen species activate p38 MAPKs to drive crypt formation in organoid cultures^45^. Together, these results suggest that p38 MAPKs play critical roles in ISC activities.

### A p38 MAPK inhibitor partially rescues villus natural aging

**Fig. 6:**
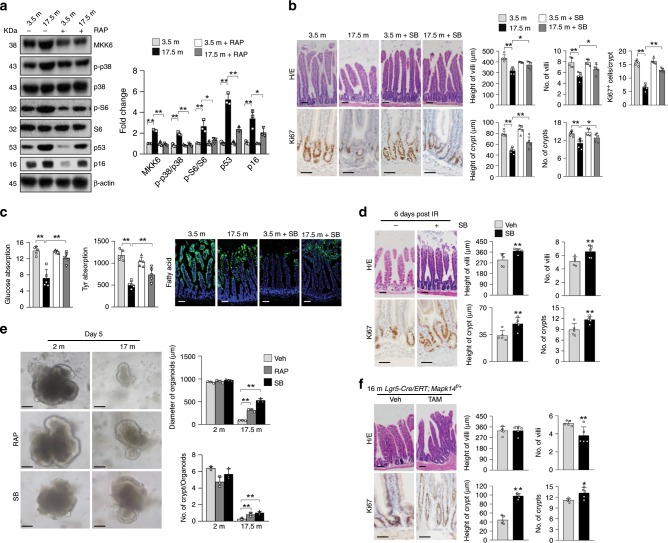
Inhibition of p38 MAPK rescues natural aging of mouse villi. **a** Western blot results showed that 17.5-month-old mouse crypt samples displayed elevated MKK6 expression, increased p38 MAPK activation, and increased p53 and p16 levels, which were suppressed by RAP treatment for 1.5 months starting at 16 months of age. Right panel: quantification data (mean ± SEM). *N* = 3 mice per group. *P < 0.05, **P < 0.01 (determined using Student’s *t* test). **b**–**d** Inhibition of p38 MAPK largely rescued the deterioration in villus and crypt structures (**b**), decreased nutrient absorption activities (**c**), and compromised regeneration (**d**) in 17.5-month-old normal mice. The mice were treated with SB203580 for 1.5 months. Data are expressed as mean ± SEM. *N* = 5 mice per group. *P < 0.05, **P < 0.01 (determined using Student’s *t* test). **e** Representative images showing that SB203580 (2 μM) or RAP (0.5 μM) partially rescued the defects in growth and crypt formation of minigut organoids isolated from 17.5-month-old mice. The inhibitors were added to the culture at day 1 of the organoid cultures. Bottom panels: quantification data (mean ± SEM). *N* = 3 mice per group. **P < 0.01 (determined using Student’s *t* test). **f** *Lgr5-CreERT;Mapk14*^*f/+*^ mice (TAM injected at 2 months of age) did not show aging-like villus structural defects at 16 months of age. The intestines were sectioned and stained with H/E or Ki67 antibodies. Right panels: quantification data (mean ± SEM). *N* = 5 mice per group. *P < 0.05, **P < 0.01 (determined using Student’s *t* test).

The above studies show that p38 MAPKs play an important role in mTORC1-driven villus/ISC premature aging. To test whether p38 MAPK plays a role in villus natural aging, we compared the crypts of 3.5- and 17.5-month-old normal mice and discovered increases in MKK6 expression and p38 activation in the old mice, which were associated with increased mTORC1 activation and were suppressed by RAP treatment (Fig. 6a). We then tested whether a small molecule inhibitor of p38 MAPK can slow villus natural aging. Administration of SB203580 to 16-month-old normal mice for 1.5 months inhibited p38 MAPK activation, manifested by decreases in phosphorylation of p38 MAPK substrate Creb (Supplementary Fig. 9f), and alleviated villus aging phenotypes, including villus structure, nutrient absorption activity, and compromised regeneration (Fig. 6b–d), as well as decreases in the number of proliferating TA cells (Fig. 6b). Inhibition of p38 MAPK has been shown to slow aging of skeletal muscle^46^. Moreover, we found that organoids derived from 17.5-month-old mice showed defects in growth and crypt formation, which were partially rescued by addition of low doses of SB203580 or RAP to the cultures (Fig. 6e), suggesting that the effects of SB203580 and RAP are directly on crypt cells. Furthermore, *Lgr5-CreERT;Mapk14*^*+/f*^ mice (TAM injected at 2 months of age) showed modest villus/crypt overgrowth but not aging-like phenotypes at 16 months of age (Fig. 6f). These results suggest that elevated p38 signaling inhibits TA cell proliferation and promotes villus natural aging.

### mTORC1 upregulates p53 and p16 expression via p38 MAPKs

How does p38 MAPK activation, downstream of mTORC1-MKK6, induce villus aging? Accumulating evidence suggests that p38 MAPK regulates aging via p53, p16, and other molecules^47–49^. Our antibody array and western blot results revealed that naturally aged crypt samples or *Tsc1*-deficient prematurely aged crypt samples showed increased levels of p53 and p16, which were suppressed by RAP treatment of the mice (Supplementary Fig. 10a, Supplementary Table 1). Moreover, aging- or mTORC1 activation-induced increases in the levels of p53 and p16 in crypt samples were suppressed by *Mapk14* deficiency or inhibition (Supplementary Fig. 10b, c). These results indicate that mTORC1 activation induces p53 and p16 expression in a p38 MAPK-dependent manner.

### p53 is critical in mTORC1-p38 MAPK-controlled villus aging

**Fig. 7:**
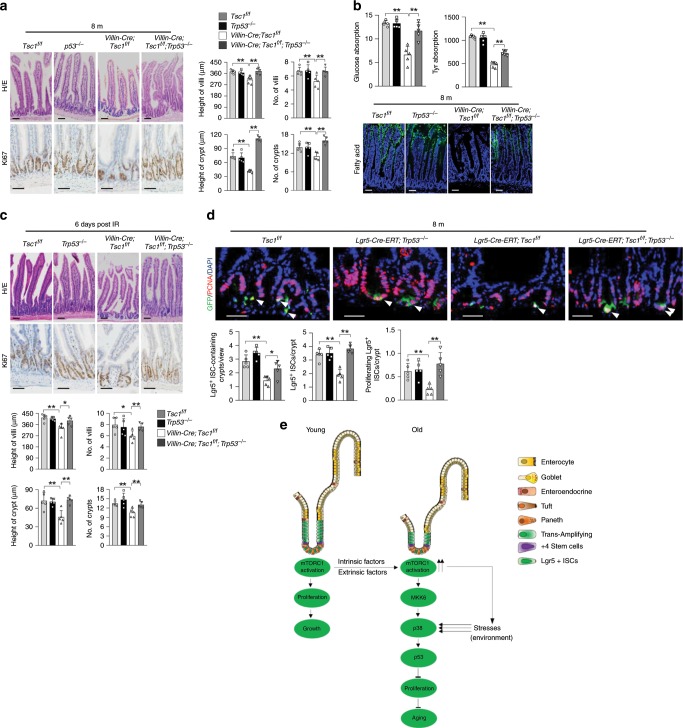
Ablation of *Trp53* prevents mTORC1-driven villus aging. **a**–**c** Deletion of *Trp53* rescued the deterioration in villus and crypt structures (**a**), decreased nutrient absorption activities (**b**), and compromised regeneration (**c**) in 8-month-old *Villin-Cre;Tsc1*^*f/f*^ mice. Data are expressed as mean ± SEM. *N* = 5 mice per group. *P < 0.05, **P < 0.0 (determined using Student’s *t* test). **d** *Trp53* ablation rescued the decrease in the numbers of ISCs and proliferating ISCs in 8-month-old *Lgr5-GFP-CreERT;Tsc1*^*f/f*^ mice. The intestinal sections were immunostained for PCNA, and cells positive for PCNA and GFP were counted and normalized to the total numbers of Lgr5^+^ ISCs. Bottom panels: quantification data (mean ± SEM). *N* = 5 mice per group. *P  < 0.05, **P < 0.0 (determined using Student’s *t* test). **e** A model for controlling gut stem and progenitor cell aging by mTORC1.

We then tested whether p53 and p16 contribute to mTORC1-driven villus aging. Although *Trp53*^*−/−*^ mice started to develop tumors at 5–6 months of age, many survived up to 7–8 months with no obvious tumor development. While 2- or 8-month-old *Trp53*^*−/−*^ mice showed normal villus structure, *Trp53* deficiency enhanced crypt growth in *Villin-Cre;Tsc1*^*f/f*^ mice and largely prevented development of villus premature aging phenotypes, including decreases in villus structure, nutrient absorption activities, increased sensitivity to IR, and decreased villus regeneration at 7 months of age (Fig. 7a–c; Supplementary Fig. 10d). We also repeated these experiments using *Lgr5-CreERT;Tsc1*^*f/f*^*;Trp53*^*−/−*^ mice and found that *Trp53* deficiency rescued the decreases in proliferating TA cells and the numbers of Lgr5^+^ ISC-containing crypts, Lgr5^+^ ISCs per crypt, and proliferating Lgr5^+^ ISCs as well (Fig. 7d). These results indicate that p53 plays an important role in *Tsc1* deletion-driven villus aging.

p16 (encoded by *Cdkn2a*) plays an important role in aging of several tissues^48,50^. However, 2- or 8-month-old *Cdkn2a*^*−/−*^ mice showed normal villus histology, and *Cdkn2a* ablation failed to affect villus growth in 2-month-old *Villin-Cre;Tsc1*^*f/f*^ mice. *Cdkn2a* deficiency showed negligible effects on aging-related histology in 8-month-old *Villin-Cre;Tsc1*^*f/f*^ mice (Supplementary Fig. 10e). These results suggest that p16 plays a lesser role than p53 in mTORC1-driven villus aging.

## Discussion

Aging-related nutrient malabsorption is a common disorder, yet the underlying etiology remains elusive. This study reveals that aging is associated with decreases in villus height and density and nutrient absorption activities, as well as increased sensitivity to IR, which may be the cause of compromised villus regeneration. These aging-related phenotypes are recapitulated in 7–8-month-old *Lgr5-CreERT;Tsc1*^*f/f*^ mice and *Villin-Cre;Tsc1*^*f/f*^ mice. Aging-related villus structural deterioration was accompanied by decreases in the number and height of crypts and the number and proliferation of ISCs. These findings, together with our observation that *Tsc1* ablation and mTORC1 activation in IECs do not affect villus cell differentiation or nutrient absorption activities in 2–3-month-old mice, suggesting that aging-related malabsorption is mainly attributable to exhaustion of gut stem cells and their reduced activity, which result in decreases in villus size and density.

While aging can be caused by complex mechanisms, we provide genetic evidence that villus and ISC aging may involve mTORC1 signaling. mTORC1 is strongly activated in ISCs and TA cells in old mice, and inhibition of mTORC1 with RAP for only 1.5 months partially restored the structure and function of intestinal villi in old mice. Moreover, *Tsc1* ablation in Lgr5^+^ ISCs leads to villus premature aging phenotypes and crypt atrophy in Paneth-less colon, which were rescued by RAP treatment. These data suggest that mTORC1 is an important regulator of gut stem cell and villus aging. Our conclusions are generally consistent with those of a recent study showing that diet restriction helps expand ISCs by inhibiting mTORC1 signaling^37^, but inconsistent with another study showing that diet-restriction-induced ISC expansion is inhibited by RAP^38^. What causes enhanced mTORC1 activation in aged IECs and ISCs? It is possible that age-related changes in systemic factors may be involved, which are now being vigorously explored^40,51,52^. These factors may include nutrients, metabolites, and growth factors^14,45^, and warrant further investigation.

Our studies also show that mTORC1 activation promotes ISC/TA cell proliferation in young mice. While mTORC1-driven cell hyperproliferation and anabolism are generally presumed to cause aging^53^, our study suggests that in the fast-turnover intestinal villi, mTORC1 activation-induced aging may not necessarily require mTORC1-induced overgrowth in earlier stages based on the following observations: (i) ablation of *Tsc1* in Lgr5^+^ ISCs at 6.5 months of age circumvents the villus overgrowth phase, which occurs at 2–3 months of age, but still induces villus premature aging; (ii) villus aging in *Tsc1-*deficient mice can be partially rescued by the administration of p38 or mTORC1 inhibitors to old mice; and (iii) *Mapk14* ablation increases crypt cell proliferation, but rescues villus premature aging. These results suggest that *Tsc1* deletion-driven ISCs exhaustion and villus aging are not directly caused by mTORC1-driven cell overproliferation.

Instead, we show that mTORC1 signaling promotes villus aging by augmenting a cell stress response pathway. mTORC1 activation increases MKK6 expression, potentiates p38 MAPK activation, and enhances p53 expression, leading to ISC exhaustion and decreases in villus size and density (Fig. 7f). Moreover, mTORC1 activation also increases cell sensitivity to IR and compromises villus regeneration in a p38 MAPK-p53-dependent manner, which is consistent with cell-based studies showing that mTORC1-activated cells are more sensitive to stress^47^. The natural function of the mTOR-MKK6-p38 MAPKs-p53 pathway may be to balance mTORC1-induced overgrowth and protect cells from runaway proliferation and oncogenic transformation, which is consistent with the concept that aging acts as an anti-hyperplasia mechanism^42^.

This study places p38 MAPK downstream of mTORC1 signaling in controlling ISC/villus aging. p38 MAPK has been implicated in the aging of skeletal muscle and other tissues^42^. We show that a p38 MAPK inhibitor can effectively improve the structure and function of naturally aged villi and that *Mapk14* ablation can prevent ISC and villus aging. These results justify p38 MAPK as an antiaging target. Since p38 MAPKs can be activated by various stress and cytokines, p38 MAPK may act as a nexus to incorporate environmental cues to influence the aging process. These insults may include mTOR activation-induced oxidative and ER/proteostatic stress, which may lead to further activation of p38 MAPKs (Fig. 7f). Our findings favor a model in which mTORC1 signaling cooperates with p38 MAPK-activating stresses to regulate aging of tissue stem cells (Fig. 7f).

Emerging evidence indicates that mTOR is a central regulator of aging that may mediate RAP- and diet restriction-induced extension of the lifespan and healthspan. While mTORC1-driven cell proliferation and incurred oxidative and proteostatic stress are generally believed to underlie cell and tissue aging, this study provides genetic evidence that mTORC1 can regulate tissue stem/progenitor cell aging and tissue aging via a prominent stress response pathway. These findings reveal a mechanism by which mTORC1 signaling regulates aging and this working model can explain the reversibility of aging.

## Methods

### Mice maintenance

*Tsc1*^*f/f*^, *Villin-Cre*, and *Trp53*^*−/−*^ mouse lines were purchased from The Jackson Laboratories. The *Lgr5-CreERT* mouse was generated in Hans Clevers’ lab. *Mapk14*^*f/f*^ was generated in Yibin Wang’s lab. *Cdkn2a*^*−/−*^ mouse was generated in Ron Depinho’s lab. These mice were maintained in SPF mouse facility of Shanghai Jiao Tong University. Most of the mice were originally in a 129/B6 background and were crossed back to B6 for four times. They can live up to 30 months in the facility. Animal experiments were carried out in accordance with recommendations in the National Research Council Guide for Care and Use of Laboratory Animals and in comply with relevant ethical regulations for animal testing and research, with the protocols approved by the Institutional Animal Care and Use Committee of Shanghai Jiao Tong University.

### Mouse drug administration and radiation

Tamoxifen (Sigma) was dissolved in corn oil, and mice were injected with 10 mg per dose daily for 3 consecutive days to activate CreERT. To inhibit mTORC1, mice were injected with RAP (3 mg/kg, Selleck, s1039) daily for the periods indicated in the study. To inhibit p38 MAPKs, mice were injected with SB203580 (5 mg/kg, Selleck, S1076) daily for the time indicated in the study. For long-term RAP or SB203580 administration, we waited at least 3 days after completion of drug treatment before doing experiments to avoid acute effects of the drugs. For ionizing radiation, mice were exposed to γ-irradiation with a dose of 5 Gy, and mice were killed 2, 3, or 6 days after IR.

### Nutrients absorption assays

The mice were fasted for 16 h before nutrient assays^54–56^. For glucose absorption test, the mice were gavaged with L-glucose solution (2 mg/g, body weight) in PBS, blood samples were collected from the tail vein at 0, 7, 15, 30, 60, 90, and 120 min, and blood glucose levels were determined, with the value at 60 min used as an indication of glucose absorption activity. For amino acid absorption test, the mice were gavaged with amino acids mixture (2 mg/g, body weight), blood samples were collected from the tail vein at different time points, and blood amino acid levels were determined with HPLC. The peak value (at 1 h) was used to represent amino acid absorption activity. For fatty acid absorption test, the mice were gavaged with BODIPY® 500/510 C1, C12 fatty acids (2 μg/g body weight, Molecular Probes #D3823), or olive oil (10 μl/g body weight). After 2 h, small intestines were excised and frozen in OCT. Ten-μm sections were cut, mounted with ProLong® Diamond Antifade Mountant with DAPI, and examined under the fluorescence microscope. In nutrient absorption studies, no significant difference was observed between male and female mice.

### Histology and immunohistochemistry

Small intestines were harvested immediately after killing and washed with PBS. The tissues (the proximal and distal jejunum and proximal colon) were fixed in 4% paraformaldehyde, embedded in paraffin, sectioned longitudinally at 4-μm, and the midline sections were stained with hematoxylin and eosin (H/E), ALP or Alcian Blue. For immunostaining, antigen retrieval was carried out by boiling the sections in citrate buffer, pH 6, for 10 min, followed by cooling for 60 min at room temperature. To eliminate endogenous peroxidases, tissues were treated in methanol containing 3% H_2_O_2_ for 30 min. Tissues were permeabilized with 0.1% Triton X-100 for 30 min. The sections were blocked in 10% goat serum for 30 min followed by primary antibodies incubation at 4 °C overnight, and secondary antibody incubation for 60 min at 37 °C. Antibodies against Ki67 (ab15580, 1:100) and Lysozyme (ab108508, 1:100) were purchased from Abcam. Antibodies against GFP (2956, 1:200), p-S6 (2211, 1:100), p-p38 (9211, 1:100), and p-Erk1/2 (9106, 1:200) were purchased from Cell Signaling Technology. Antibodies against PCNA (sc-56, 1:100) were purchased from Santa Cruz Biotechnology.

### Antibody array analysis

To compare the activation and expression of proteins involved in cell proliferation, we chose to use snap-frozen small intestine samples of wild-type and *Villin-Cre; Tsc1*^*f/f*^ mice since isolated crypts by enzyme digestion at 37 °C produced inconsistent results. The samples were analyzed with phospho-Exploerer (PEX100) by the H-Wayen Biotechnologies.

### Crypt isolation and culture and GFP^+^ ISCs sorting

To isolate crypts, the small intestine, mostly jejunum and ileum, was removed immediately, and the stool was flushed out with ice-cold PBS. The tissues were dissected and opened longitudinally and cut into 1 -cm pieces, which were placed in PBS with 2 mmol/L EDTA for 30 min at 4 °C and then in PBS with 54.9 mmol/L d-sorbitol and 43.4 mmol/L sucrose. The pieces were then vortexed for 1–2 min, filtered by a 70 μm sterile cell strainer, and enriched by centrifugation at 150 g for 10 min at 4 °C. Nearly 500 crypts were suspended in 50 μL growth factor reduced phenol-free Matrigel (BD Biosciences). Afterward, a 50 μL droplet of Matrigel/crypt mix was put in the center well of a 12-wells plate and after 30 min of polymerization, 650 μl of minigut medium was overlain. Minigut medium (advanced DMEM/F12 supplemented with HEPES, l-glutamine, N2, and B27) was added to the culture with R-Spondin, Noggin, and EGF. The medium was changed every 2–3 days. For GFP^+^ ISCs isolation, we first isolated crypts and digested them at 37 °C for 5 min with 0.25% trypsin, centrifuged at 150–200 g for 3 min, and resupensed in 0.1% BSA in ice-cold PBS. We purified GFP^+^ cells with FACS sorting and collected. These cells were resupensed in PBS, smeared onto coated slides, dried at 4 °C. The slides were then fixed in 4% PFA, and GFP^+^ cells were countered under the fluorescence microscope.

### Epithelial cell isolation and culture

For primary IECs isolation, intestines were opened longitudinally and washed in Ca^2+^ and Mg^2+^-free Hanks’ balanced salt solution (HBSS) containing 2% glucose, 25 ng of amphotericin B/ml, 100 U of penicillin/ml, and 100 mg of streptomycin/ml, which were cut into 1-mm fragments and incubated for 10 min at 22 °C on a shaker platform in HBSS containing collagenase XIa (Sigma), 2% bovine serum albumin, and 0.2 mg of soybean trypsin inhibitor/ml (Sigma). Cells and small sheets of intestinal epithelium were separated from the denser intestinal fragments by harvesting supernatants after two 60-s sedimentations in medium containing Dulbecco’s modified Eagle medium (DMEM) (Gibco), 10% sorbitol (Gibco), 100 U of penicillin/ml, 100 mg of streptomycin/ml, and 5% fetal bovine serum. Cells were centrifuged five times at 1200 *g* for 3 min in DMEM plus 2% sorbitol. Supernatants were discarded, and the cells were cultured in 10-cm plates coated with 40 ml of Matrigel per cm^2^ diluted 1:2 in phenol-red-free DMEM (Sigma).

### S^35^-methionine incorporation assay

Primary IECs were pre-incubated with DMEM without methionine and cysteine for 30 min, and then labeled with EasyTag™ EXPRESS ^35^S Protein Labeling Mix (NEG772007MC) for 1 h. To determine the rates of protein synthesis, cell extracts were subjected to immunoprecipitation with individual antibodies. Antibodies against MKK6 (9264, 1:250) were purchased from Cell Signaling Technology. Antibody against Gapdh (sc32233, 1:250) was purchased from Santa Cruz Biotechnology. The immunoprecipitates were eluted and subjected to SDS-PAGE, and the gel was dried and visualized with phosphoimaging.

### Western blot analysis

For *Villin-Cre; Tsc1*^*f/f*^ mice, villus tissues were scraped from the small intestines. For *Lgr5-CreERT; Tsc1*^*f/f*^ mice, crypts were isolated. In these experiments, the mice were starved overnight and harvested in the following morning. These samples were lysed in T-PER™ Tissue protein extraction reagent purchased from Thermo Scientific (78510) containing 1 mM PMSF and 1 μg/ml aprotonin, leupeptin, and pepstatin. Cells were lysed in TNEN buffer containing 1 mM PMSF and 1 μg/ml aprotonin, leupeptin, and pepstatin. The protein concentration was determined by a Bio-Rad assay. Proteins were resolved by SDS-PAGE and transferred to polyvinylidenedifluoride membranes (Millipore). Antibodies against MKK6 (9264, 1:1000), p-p38 (9211, 1:1000), p38 (9212, 1:1000), p-Erk1/2 (9106, 1:1000), Erk1/2 (9102, 1:1000), cyclin E (4129, 1:1000), p53 (2524, 1:1000), p-S6 (1211, 1:1000), p-p70S6K (9234, 1:1000), p-4E-BP1 (2855, 1:1000), 4E-BP1 (9644, 1:1000), p-mTOR (5536, 1:1000), and mTOR (2983, 1:1000) were purchased from Cell Signaling Technology. Antibodies against β-actin (sc81178, 1:1000), p16 (sc1207, 1:1000) and PCNA (sc-56, 1: 1000) were purchased from Santa Cruz Biotechnology. The protein bands were quantitated using the software provided by FluoChem M system (Protein Simple). Uncropped western blot images can be found in Supplementary Fig. 11.

### MKK6 knockdown

Silencer pre-designed siRNA targeting mouse MKK6 was purchased from Gene Pharma (74366, 74367, 74368), which were transfected into*Tsc1*^*+/+*^ and *Tsc1*^*−/−*^ MEFs following the manufacturer’s protocol. Knockdown efficiency was determined by western blot.

### Quantitative PCR

The total RNA was extracted from cells, crypt, or villi tissues with TRIzol reagent (Invitrogen) following the manufacturer’s protocol. Complementary DNA was synthesized with 0.5 μg of the total RNA using Transcriptor First Strand cDNA Synthesis Kit (Roche). The detection and quantification of target mRNA were performed with real-time PCR, which was normalized to the levels of β-actin. Real-time PCR was carried out using the Applied Biosystems 7500 system.

The following primers were used in this study: *Mapk14* forward primer: 5′-GGCACCTGCCATCAGTAGTT-3′, reverse primer: 5′-CCAGGGCTTCCAGAAGACAG-3′; *Mkk6* forward primer: 5′-GGCACCTGCCATCAGTAGTT-3′, reverse primer: 5′-CCAGGGCTTCCAGAAGACAG-3′.

### Villi and crypt quantitation

The numbers of villi per view (10 × 10 magnification) were counted double blindly. The height of villi and crypts were measured from the top to the bottom using NIKON-TiuNIS-Elements microscope. Each group has at least three mice, and the results represent the average of five sections per mouse.

### Statistical analysis

The data are expressed as the mean with the standard error of the mean (±SEM) or as mean with standard deviation (±SD), if replicates are used. The number of samples (*n*) is indicated in the figure legends. Unpaired two-tailed Student’s *t* test was applied to determine the significance of the differences between two groups and *P*-value < 0.05 was considered as statistically significant.

### Reporting summary

Further information on research design is available in the Nature Research Reporting Summary linked to this article.

## Supplementary information

Supplementary Information

Peer Review File

Reporting Summary

## Data Availability

The source data for Figs. 1a–e, g, h, 2a–d, 3a–d, 4a–e, 5a–d, 6a–f, 7a–d and Supplementary Figs. 1a, d, 2a–c, h, 3b, c, 4a, 5b, d, f, g, 6b, c, 7b–d, 8b, 9b, c, 10d, e are provided as a Source Data file. The remaining data are contained within the article, supplementary information, or available from the authors upon request. A reporting summary for this article is available as a Supplementary Information file.

## Source Data

Source Data
