# Supplementary material for: Gut stem cell aging is driven by mTORC1 via a p38 MAPK-p53 pathway
Source: Nat Commun. 2020 Jan 2;11:37. doi: 10.1038/s41467-019-13911-x (PMC6940394; doi:10.1038/s41467-019-13911-x)
Supplement: Supplementary file 1 — Supplementary Information [file 41467_2019_13911_MOESM1_ESM.pdf]

Supplementary Information

Gut stem cell aging is driven by mTORC1 via a p38 MAPK-p53 pathway

Dan He et al.

Supplementary Figure 1

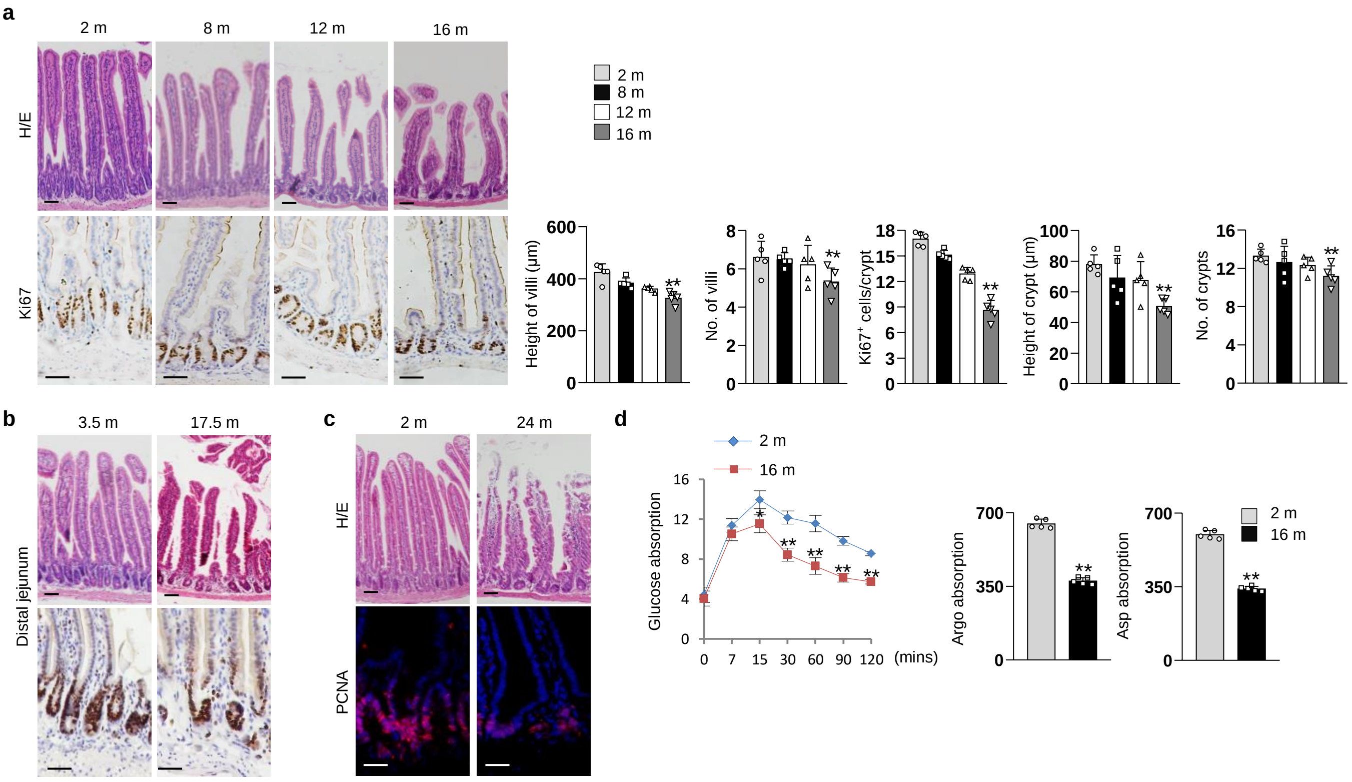

Supplementary Figure 1. Old mice showed defects in villus/crypt structure and nutrient absorbing activities.
(a). Comparison of villus structure and TA cell proliferation of 2-, 8-, 12-, and 16-month old mice. The proximal jejunum samples were sectioned and stained with H/E or Ki67 antibodies. Right panels: quantitation data (mean ± SEM). N=5 mice per group. **p<0.01 (determined using Student's *t*-test). (b). Comparison of the distal jejunum of 3.5 and 17.5-month old mice. (c). Comparison of the proximal jejunum of 3.5 and 24-month old mice. (d). Sixteen-month-old mice showed a decrease in nutrient absorbing activities for glucose and amino acids (arginine and aspartic acid). Data are expressed as mean ± SEM. N=5 mice per group. *p<0.05, **p<0.01 (determined using Student's *t*-test).

Supplementary Figure 2

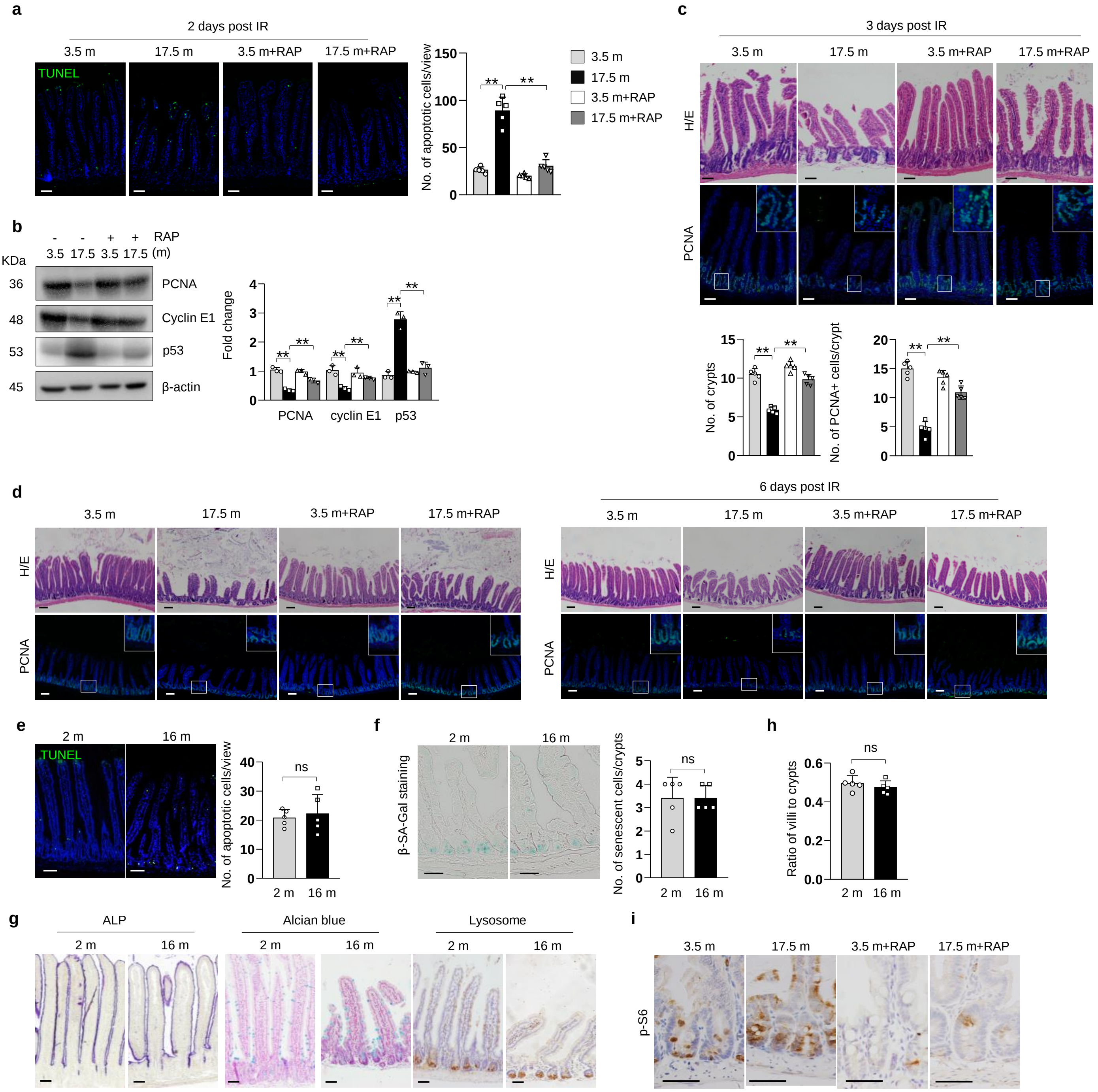

Supplementary Figure 2. mTOR activation increases sensitivity to IR in villi/crypts of geriatric mice.
(a). Seventeen and half-month-old mice showed an increase in apoptotic cells 2 days after IR, which was rescued by RAP. Right panels: quantification data (mean ± SEM). N=5 mice per group. **p<0.01 (determined using Student's *t*-test). (b). The intestine samples of 17.5-month-old mice showed decreases in PCNA and cyclin E and an increase in p53 in crypt samples at day 2 after IR, which was rescued by RAP. Right panel: quantitation data (mean ± SEM). N=5 mice per group. ** p<0.01 (determined using Student's *t*-test). (c). Seventeen and half-month-old mice showed increased sensitivity to IR-induced decreases in the numbers of crypts and proliferating cells compared to young mice at day 3 post IR, which were partially rescued by 1.5 months of RAP treatment (3 mg/kg body weight) starting at 16 months of age. Bottom panels: quantitation data (mean ± SEM). N=5 mice per group. **p<0.01 (determined using Student's *t*-test). (d). Lower magnification images of intestinal sections of 3.5 and 17.5-month-old mice (left panel) and villus regeneration in these mice at day 6 post IR (right panel). (e-h). No difference was observed between 2- and 16-month-old mice in the percentage of apoptotic cells (e) or SA-β-Gal senescent cells (f) in the villi, differentiation of villus cells (g), or the villus-to-crypt ratios (h). N=5. ns, not significant. (i). mTORC1 activation was inhibited by RAP treatment. The 16-month-old mice were sacrificed after 1.5 months of RAP treatment and the proximal jejunum sections were stained for p-S6.

Supplementary Figure 3

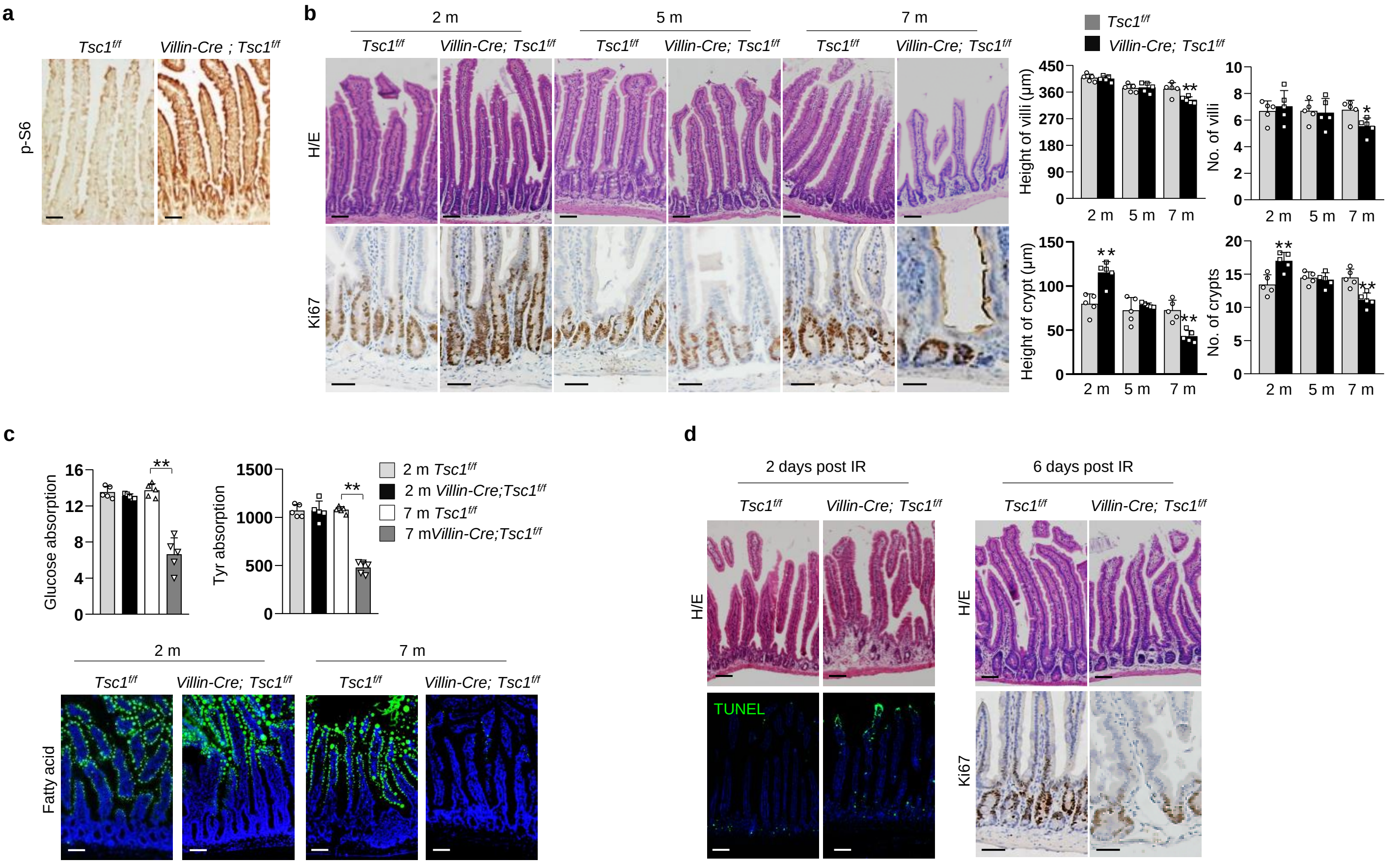

Supplementary Figure 3. Ablation of *Tsc1* in enterocytes induces villus premature aging.
(a). Immunostaining of p-S6 showed that *Villin-Cre; Tsc1$^{f/f}$* mouse intestine sections displayed enhanced mTORC1 activation. (b). Two-month-old *Villin-Cre; Tsc1$^{f/f}$* mice showed a villus/crypt overgrowth phenotype, 5-month-old mutant mice showed villi similar to age-matched wild type mice, and 7-month-old mutant mice showed aging-like change in villus structure. Right panels: quantitation data (mean ± SEM). N=5 mice per group. *p<0.05, **p<0.01 (determined using Student's *t*-test). (c). *Villin-Cre; Tsc1$^{f/f}$* mice showed defects in nutrient absorption activities at 7 months of age. Data are expressed as mean ± SEM. N=5 mice per group. **p<0.01 (determined using Student's *t*-test). (d). *Villin-Cre; Tsc1$^{f/f}$* mice showed increased sensitivity to IR and villus regeneration defects. The 2-month-old mice were radiated at 5.0 Gy and were sacrificed 2 or 6 days later. Intestinal sections were stained with H/E, TUNEL, or anti-Ki67 antibodies.

Supplementary Figure 4

a

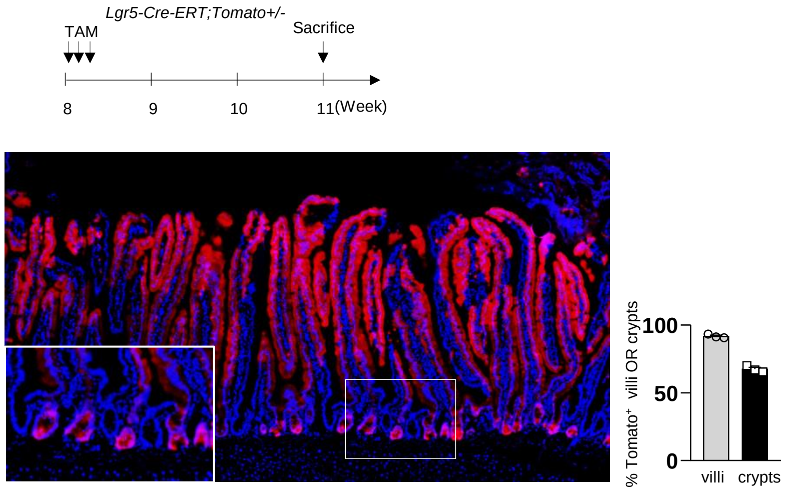

b

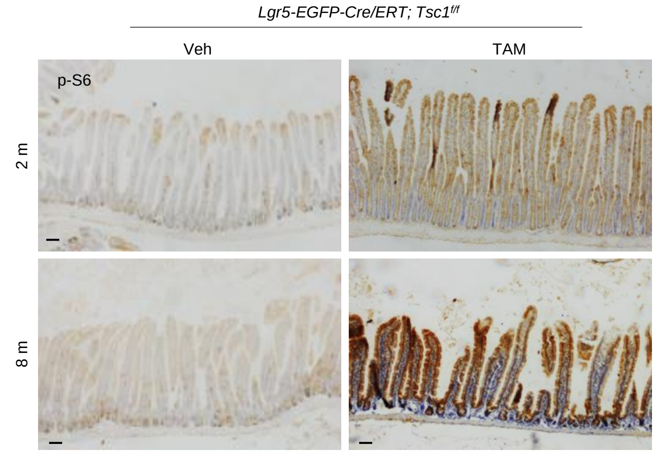

Supplementary Figure 4. Labelling of villi in *Lgr5-CreERT; tdTomato* mice and mTORC1 activation in *Lgr5-CreERT; Tsc1$^{f/f}$* mice.

(a). Tracing with *Lgr5-CreERT; tdTomato* mice revealed that almost all villi are labeled. One-month-old mice were administrated 3 daily doses of TAM and the mice were euthanized at 2 months of age. Right panel: quantitation data. N=3.
(b). Immunostaining of p-S6 revealed that *Lgr5-Cre/ERT; Tsc1$^{f/f}$* mouse intestine sections showed enhanced mTORC1 activation 1 or 7 months after TAM treatment in almost all villi.

Supplementary Figure 5

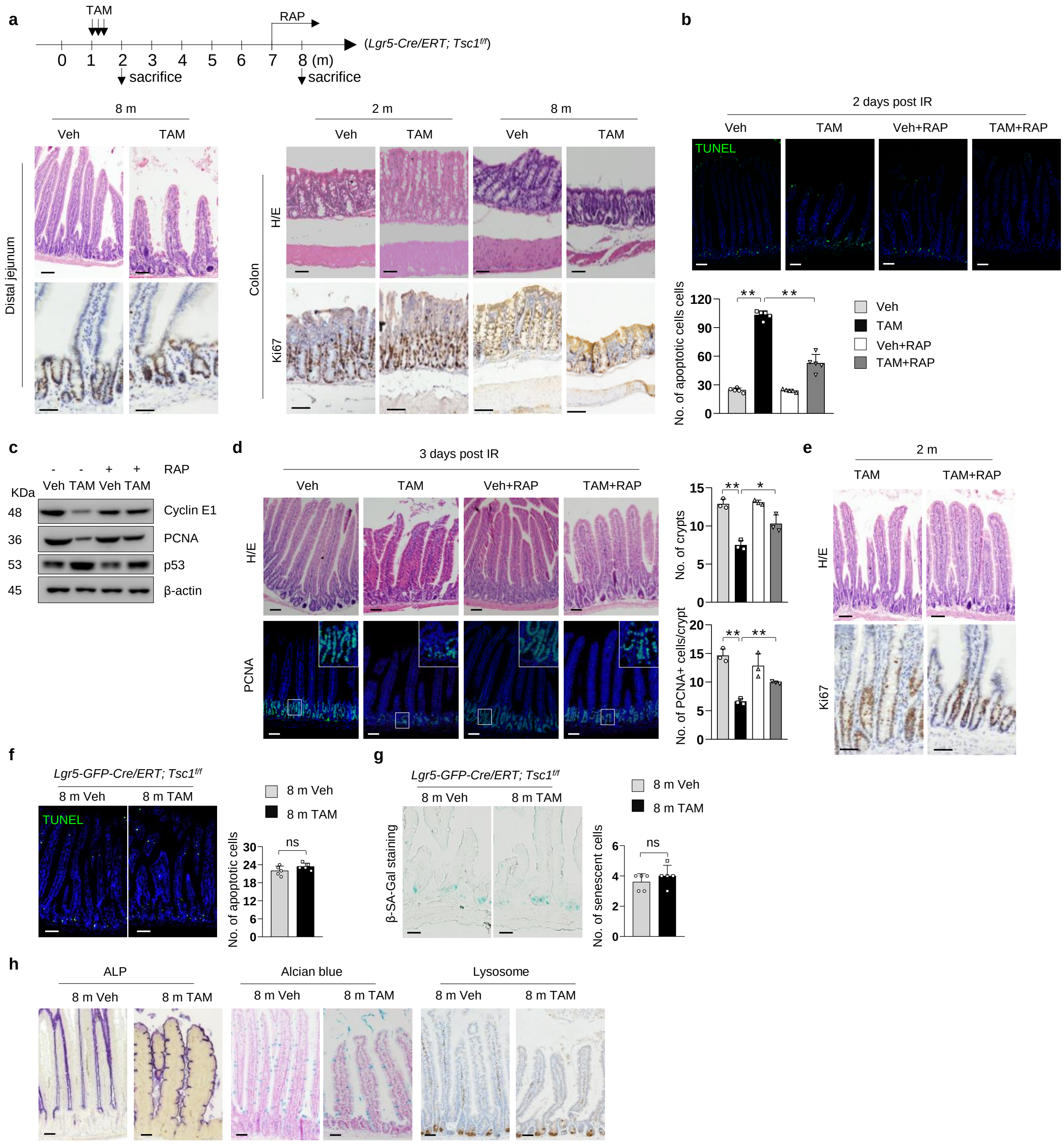

Supplementary Figure 5. Ablation of *Tsc1* in ISCs induces villus premature aging.

(a). H/E staining revealed that *Lgr5-Cre/ERT; Tsc1$^{f/f}$* mice showed aging of distal jejunum at 8 months of age and overgrowth of colon crypts at 2 months of age and atrophy at 8 months of age. (b). *Lgr5-Cre/ERT; Tsc1$^{f/f}$* mice showed an increase in apoptotic cells on intestinal sections 2 days after IR, which was rescued by RAP. Bottom panels: quantification data (mean ± SEM). N=5 mice per group. **p<0.01 (determined using Student's *t*-test). (c). The intestine samples of *Lgr5-Cre/ERT; Tsc1$^{f/f}$* mice showed decreases in PCNA and cyclin E and an increase in p53 in crypt samples at day 2 after IR, which was rescued by RAP. (d). *Lgr5-Cre/ERT; Tsc1$^{f/f}$* mice showed increased sensitivity to IR-induced decreases in the numbers of crypts and proliferating cells at day 3 post IR. Right panels: quantification data (mean ± SEM). N=3 mice per group. *p<0.05, **p<0.01 (determined using Student's *t*-test). (e). RAP treatment for 1 month recued the overgrowth of crypts in young *Lgr5-CreERT; Tsc1$^{f/f}$* mice. (f-h). The villi of 8-month-old *Lgr5-Cre/ERT; Tsc1$^{f/f}$* mice (TAM injected) showed no change in the number of apoptotic cells (f), β-SA-Gal positive senescent cells (g), or differentiation of villus cells (h). N=5. ns, not significant.

Supplementary Figure 6

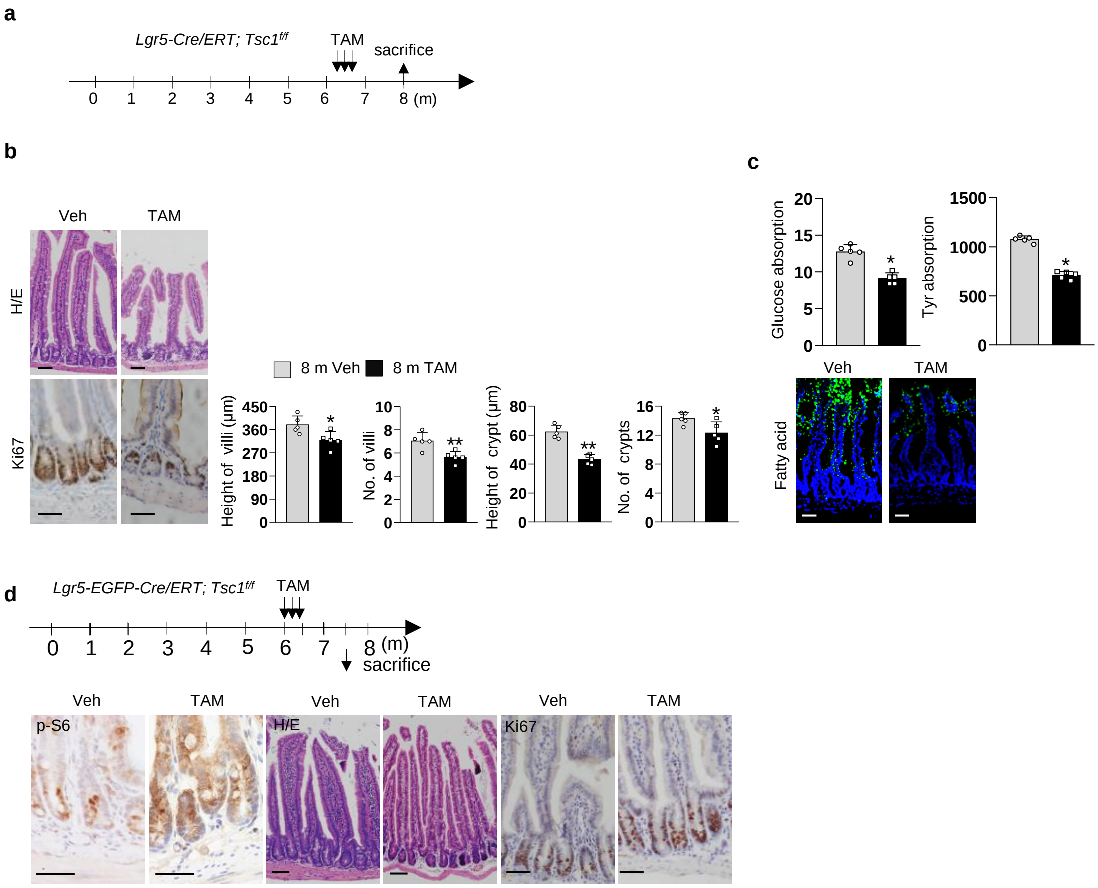

<u>Supplementary Figure 6. Ablation of *Tsc1* in in 6.5-month-old mice induces villus premature aging.</u>
(a). Diagram showing the time for TAM administration (for b and c). (b,c). Ablation of *Tsc1* in 6.5-month-old mice induced defects in villus and crypt structure (b) and decreased nutrient absorption activities at 8 months of age (c). Right panels for (b): quantification data (mean ± SEM). N=5 mice per group. *p<0.05, **p<0.01 (determined using Student's *t*-test). (d). H/E staining revealed that administration of TAM to 6.5-month-old *Lgr5-Cre/ERT; Tsc1$^{f/f}$* mice did not result in overgrowth of the crypt at 7.5 months of age.

Supplementary Figure 7

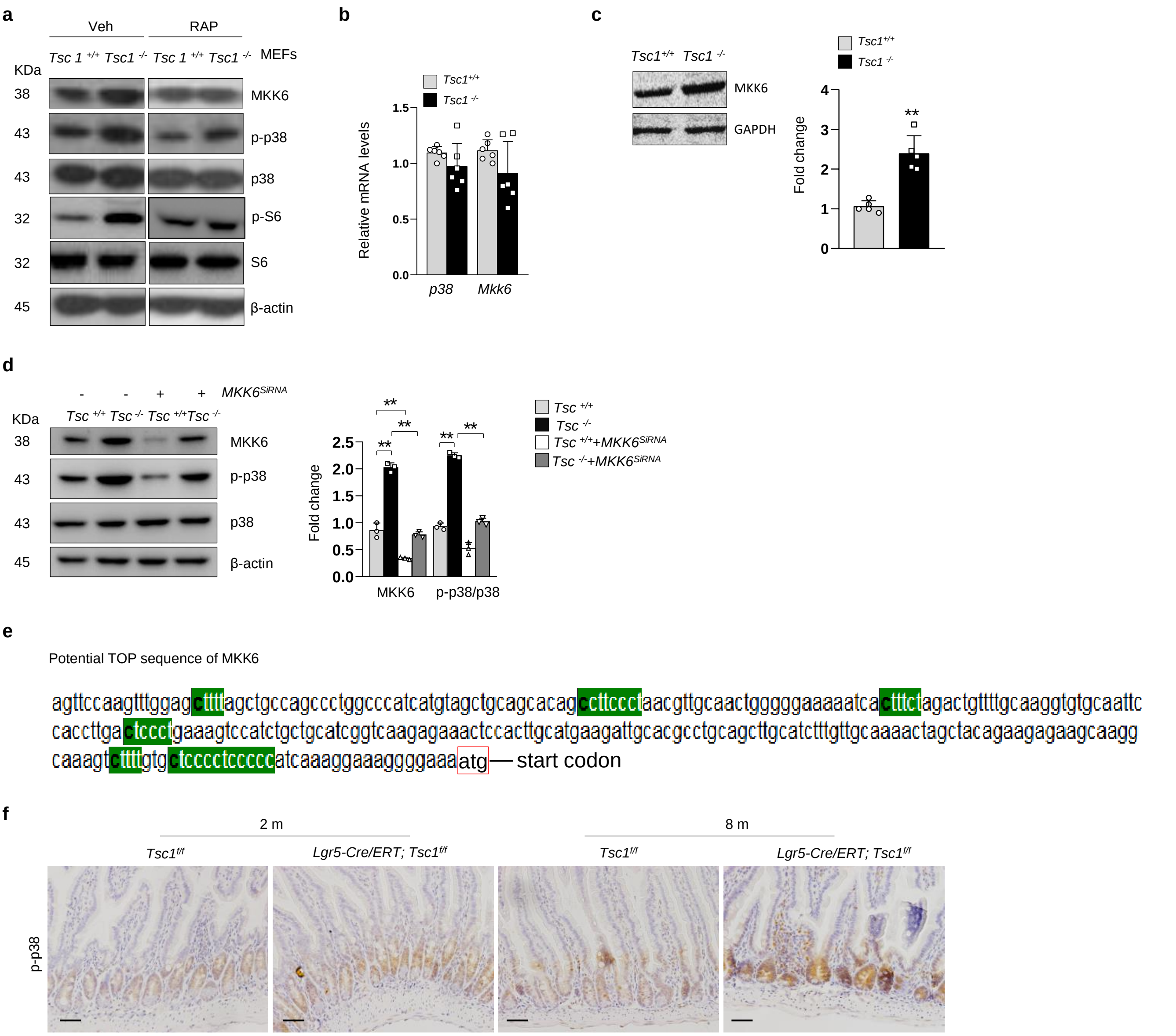

<u>Supplementary Figure 7. *Tsc1* deficiency leads to increased expression of MKK6 and activation of p38 MAPKs.</u>
(a). Western blot results revealed that *Tsc1*$^{-/-}$ MEFs showed an increase in expression of MKK6 and activation of p38 MAPKs, which were suppressed by RAP. Primary MEFs were derived from *Tsc1*$^{f/f}$ mice, infected with Cre-expressing retroviruses, selected for 3 days with puromycin, and then used for western blot analysis. (b). Quantitative PCR results showed that *Tsc1* deficiency did not significantly alter the mRNA levels of *Mkk6* in MEFs. The mRNA level of *Mkk6* of wild type cells was set at 1.0. N=6. (c). *Tsc1* deficiency increased the levels of radio-labelled MKK6 protein in MEFs. Right panel: quantitation data (mean ± SD). N=5 repetitive experiments. **p<0.01 (determined using Student's *t*-test). (d). Western blot results showed that knockdown of MKK6 led to a decrease in p38MAPK activation in *Tsc1* deficient MEFs. Right panel: quantitation data (mean ± SD). N=3 repetitive experiments. ** p<0.01, determined using Student's *t*-test. (e). Sequences of the mouse *Mkk6* 5' untranslated region. The putative TOP sequences were highlighted. (f). Immunostaining revealed that activation of p38 MAPKs was further enhanced in crypt cells of 8-month-old *Lgr5-Cre/ERT; Tsc1*$^{f/f}$ mice (TAM injected). The proximal jejunum sections were stained for p-p38 MAPKs.

Supplementary Figure 8

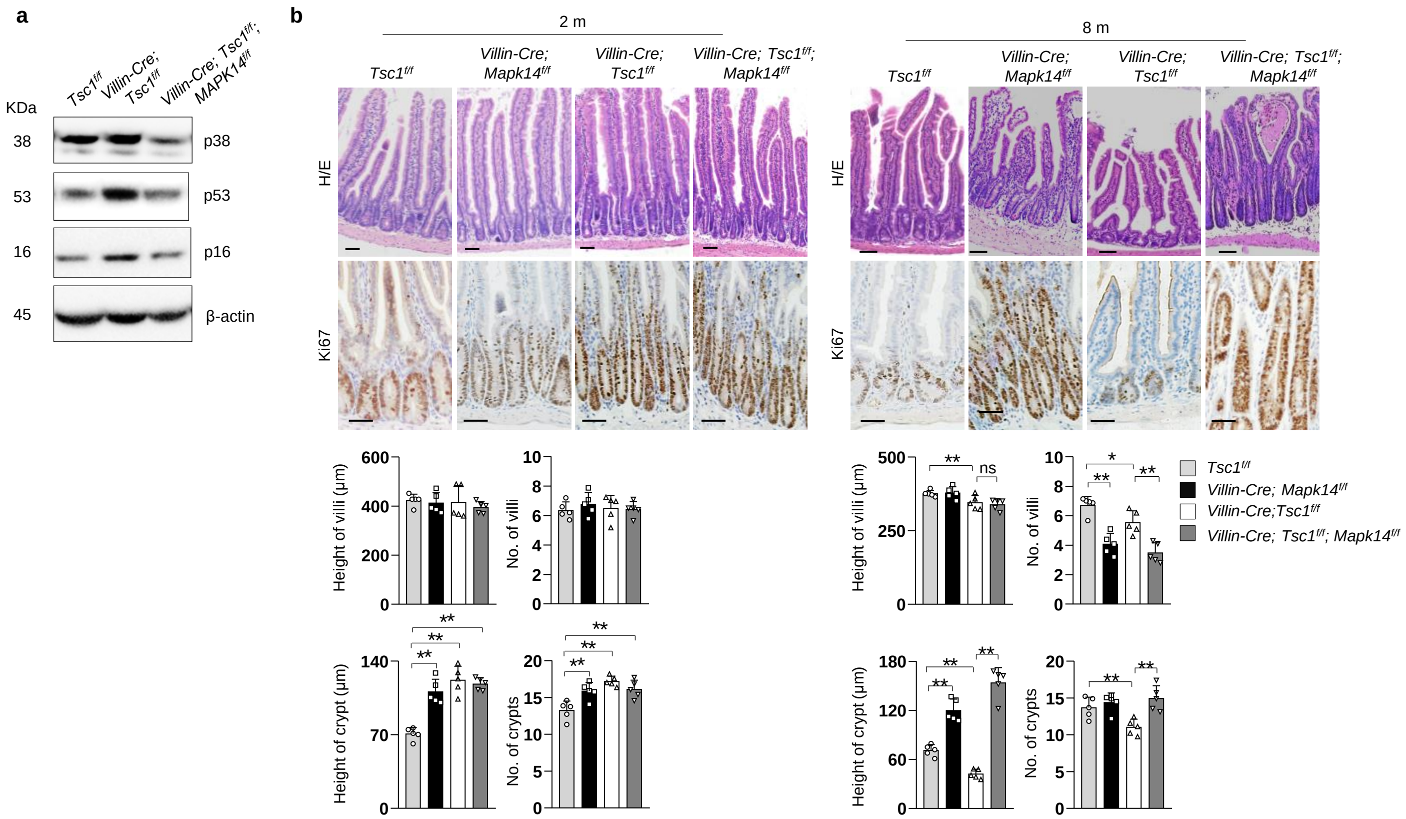

Supplementary Figure 8. p38 MAPKs mediate the pro-aging effects of mTORC1 in *Villin-Cre; Tsc1$^{f/f}$* mice.

(a). Western blot analysis revealed that p38α was largely deleted in small intestine samples of *Villin-Cre; Mapk14$^{f/f}$* mice. (b). *Mapk14* deficiency rescued villus premature aging and led to overgrowth of villus/crypt in *Villin-Cre; Tsc1$^{f/f}$* mice at 8 months of age. The decrease in the number of villi was due to fusion of the villi. Bottom panels: quantitation data (mean ± SEM). N=5 mice per group. **p<0.01 (determined using Student's *t*-test).

Supplementary Figure 9

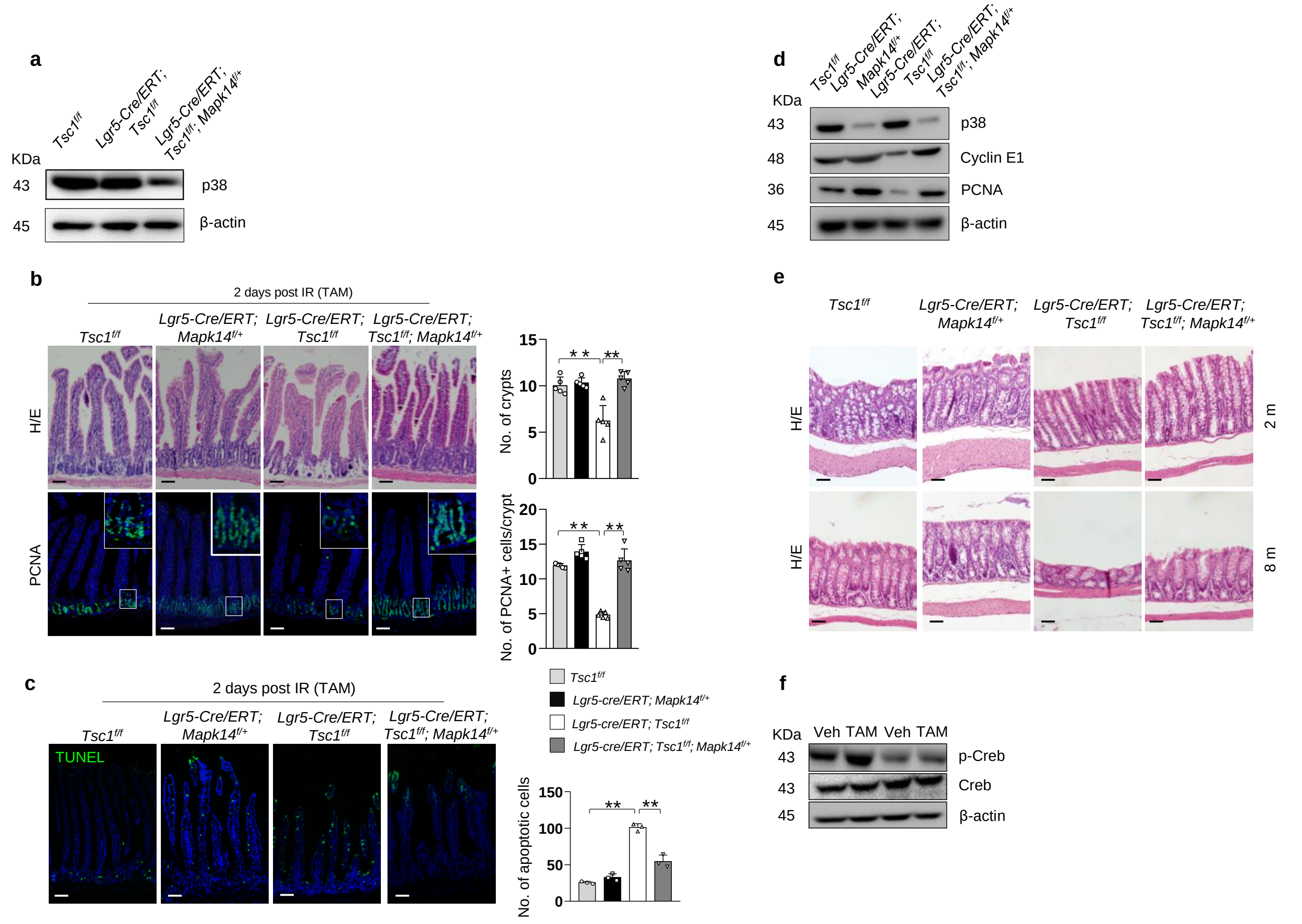

Supplementary Figure 9. p38 MAPKs mediate the pro-aging effects of mTORC1 in *Lgr5-CreER; Tsc1$^{f/f}$* mice.

(a). Western blot analysis revealed that p38α was deleted in crypt samples of *Lgr5-CreERT; Mapk14$^{f/+}$* mice. (b). *Mapk14* haplo-deficiency rescued the increased sensitivity to IR-induced decreases in the numbers of crypts and proliferating cells at day 2 post IR. Right panels: quantitation data (mean ± SEM). N=5 mice per group. **p<0.01 (determined using Student's *t*-test). (c). *Mapk14* haplo-deficiency rescued the increase in apoptotic cells 2 days after IR in *Lgr5-CreERT; Tsc1$^{f/f}$* mice. Bottom panels: quantitation data (mean ± SEM). N=3 mice per group. **p<0.01(determined using Student's *t*-test). (d). *Mapk14* haplo-deficiency rescued the decreases in PCNA and cyclin E and increase in p53 in crypt samples of *Lgr5-Cre/ERT; Tsc1$^{f/f}$* mice. (e). *Mapk14* haplodeficiency partially rescued colon crypt atrophy of *Lgr5-Cre/ERT; Tsc1$^{f/f}$* mice at 8 months of age. (f). SB203580 (SB) inhibited p38 MAPK activation, with p-Creb as an indicator. The mice were treated with SB203580 daily.

Supplementary Figure 10

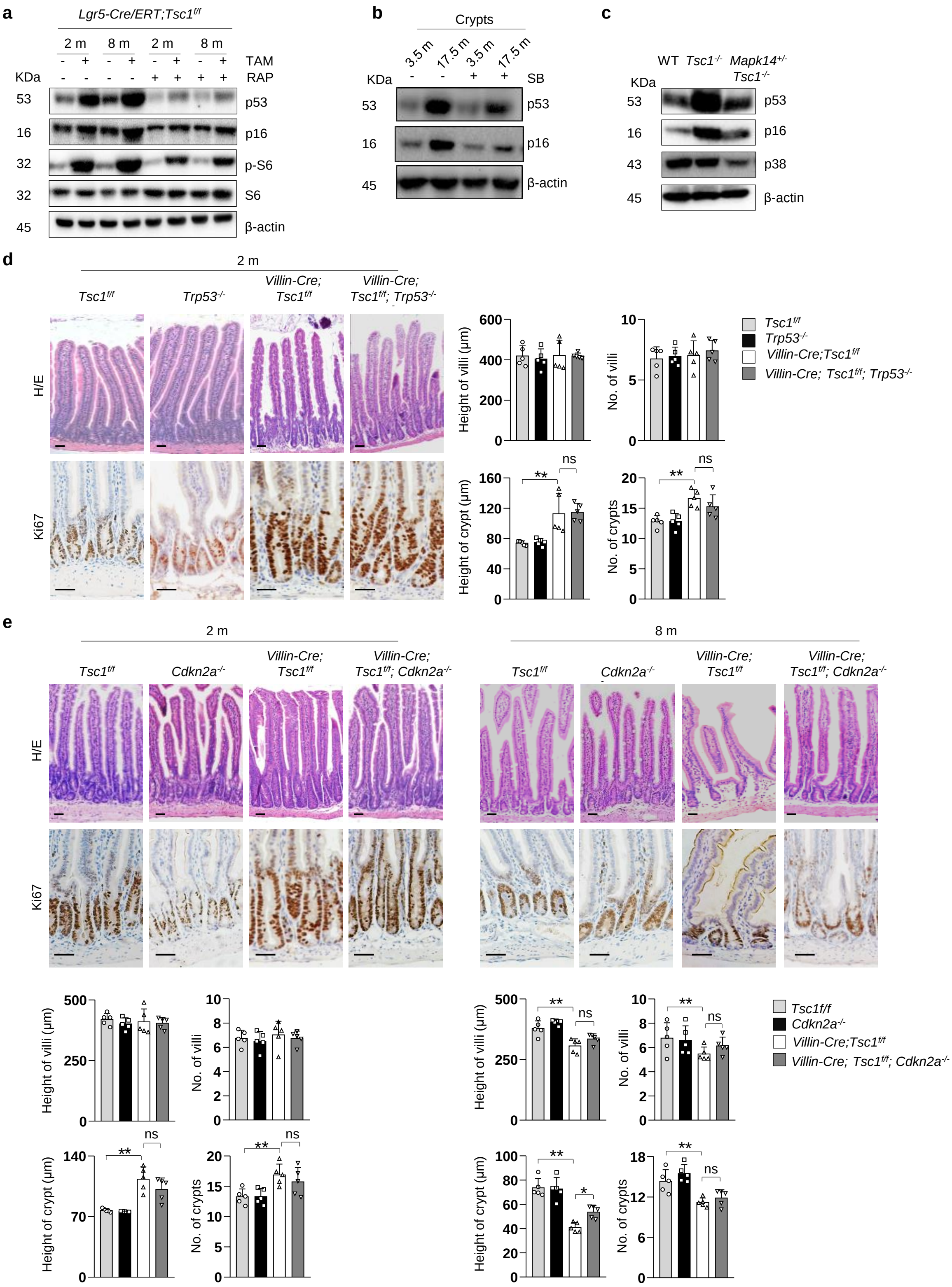

Supplementary Figure 10. The different effects of p53 and p16 on mTORC1-driven villus aging.
(a). Western blot results showed that *Tsc1* deletion induced an increase in the protein levels of p53 and p16 in crypt samples, which were suppressed by RAP. (b). Western blot results revealed that 17.5-month-old mice showed an increase in the levels of p53 and p16 in the crypts, which were suppressed by treating the mice with SB203580. (c). *Mapk14* haplo-deficiency suppressed the elevation of p53 and p16 in crypt samples of *Lgr5-CreERT; Tsc1$^{f/f}$* mice. (d). H/E staining of intestine sections of 2-month-old *Villin-Cre; Tsc1$^{f/f}$*, *Trp53$^{-/-}$*, and *Villin-Cre; Tsc1$^{f/f}$; Trp53$^{-/-}$* mice. Right panels: quantitation data (mean ± SEM). N=5. **p<0.01 (determined using Student's *t*-test). (e). H/E staining of intestine sections of 2- and 8-month-old *Villin-Cre; Tsc1$^{f/f}$*, *Cdkn2a$^{-/-}$* mice, and *Villin-Cre; Tsc1$^{f/f}$; Cdkn2a$^{-/-}$* mice. Bottom panels: quantitation data (mean ± SEM). N=4 mice per group. *p<0.05, **p<0.01 (determined using Student's *t*-test).

Supplementary Figure 11. Uncropped western blot images.

Fig. 1g

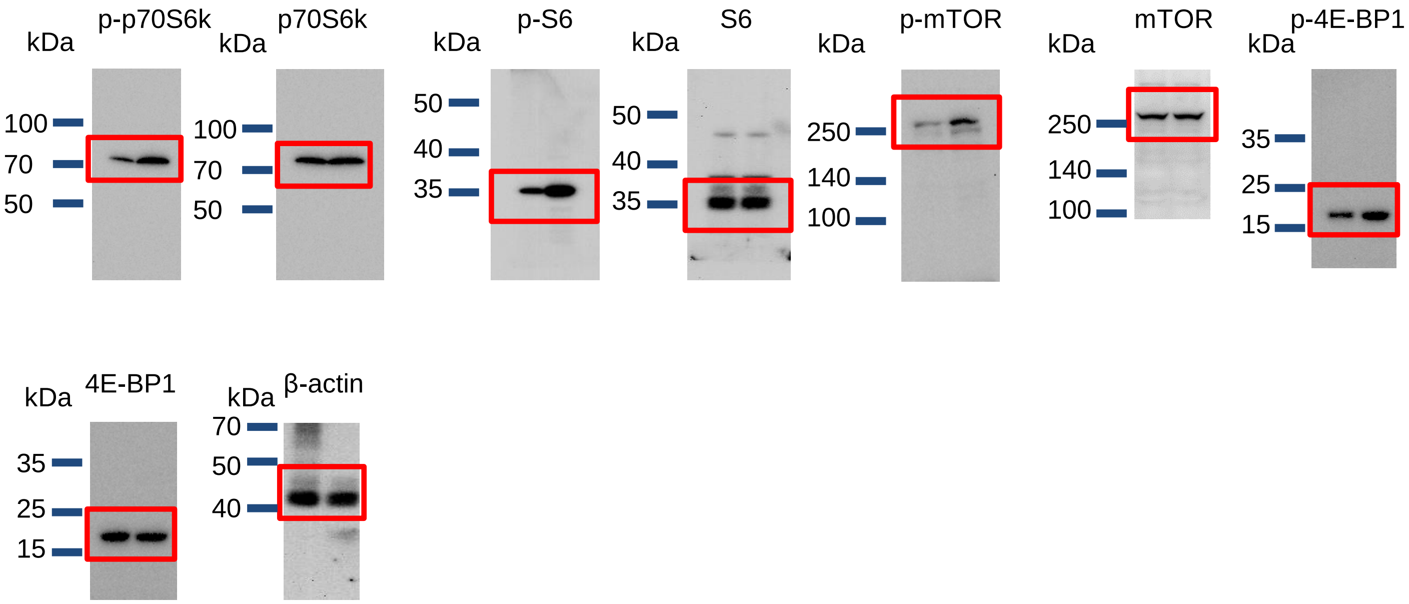

Fig. 4a

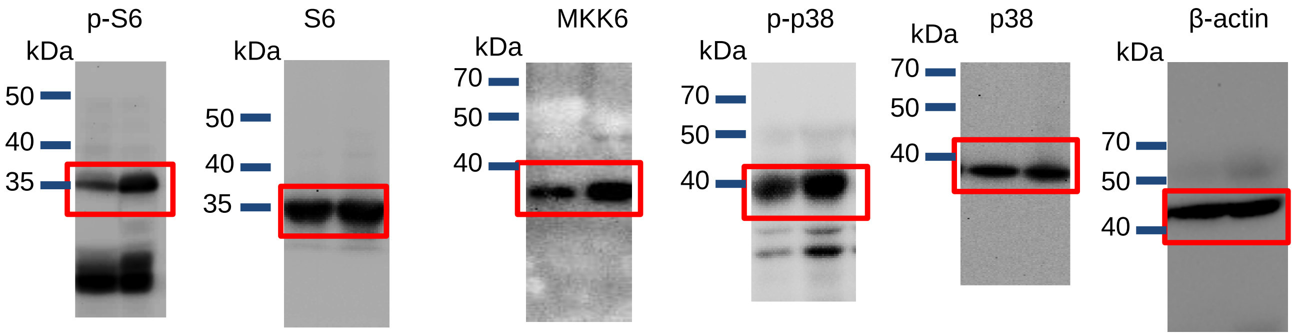

Fig. 4b

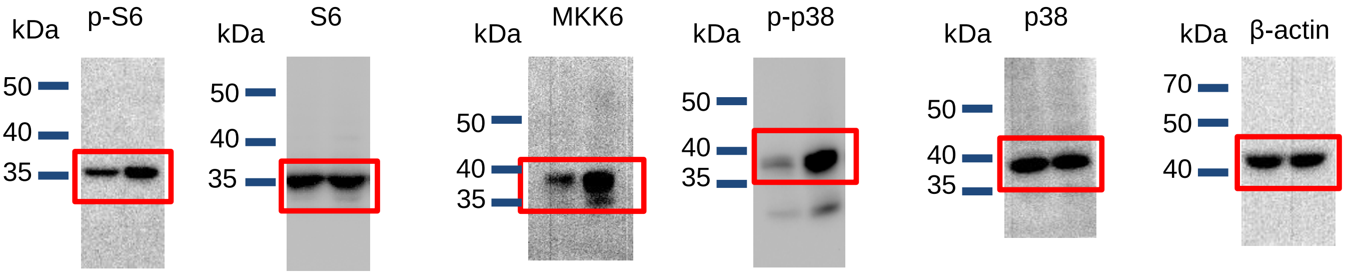

Fig. 4d

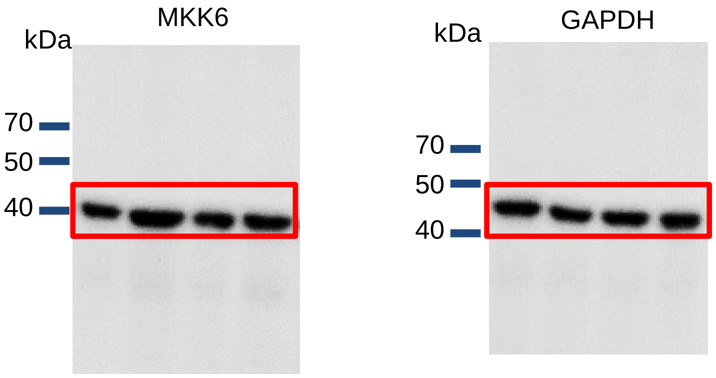

Fig. 4e

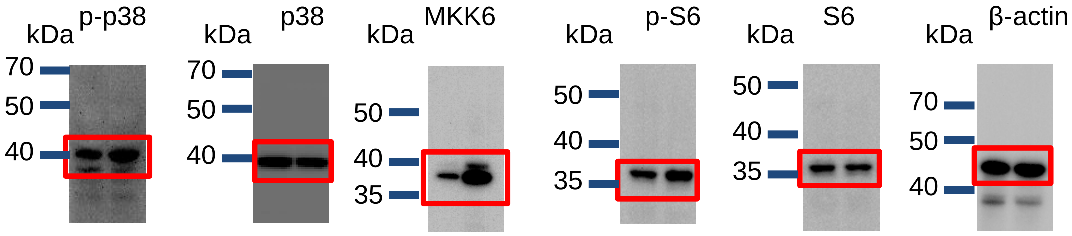

Fig. 6a

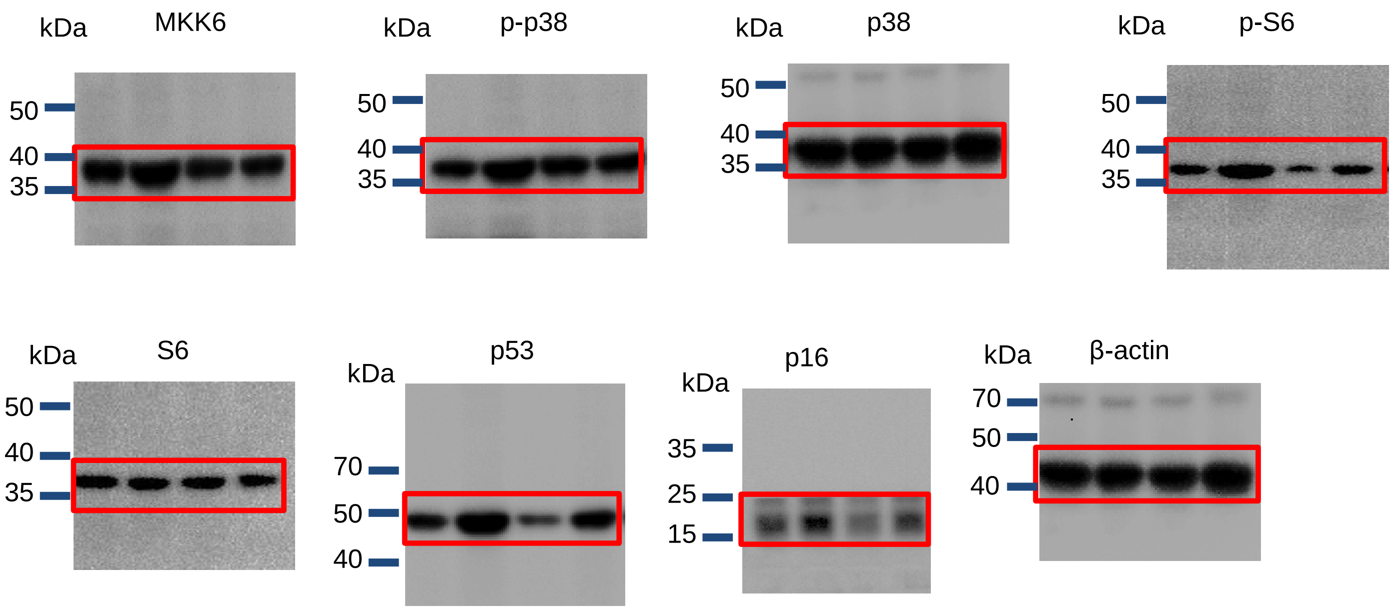
kDa
MKK6
50
40
35
kDa
p-p38
50
40
35
kDa
p38
50
40
35
kDa
p-S6
50
40
35
kDa
S6
50
40
35
kDa
p53
70
50
40
kDa
p16
35
25
15
kDa
β-actin
70
50
40

Supplementary Fig. 2b

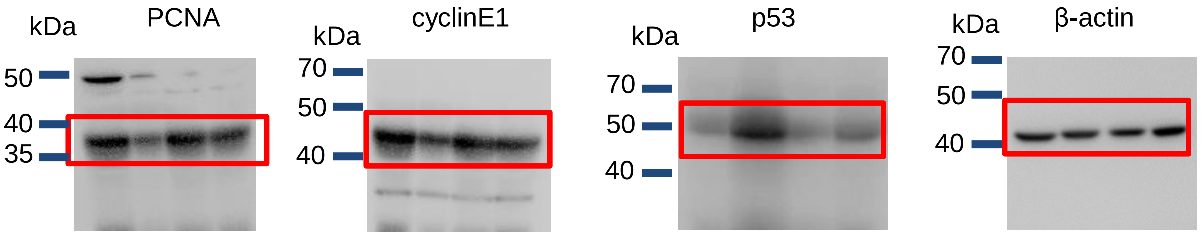

Supplementary Fig. 5c

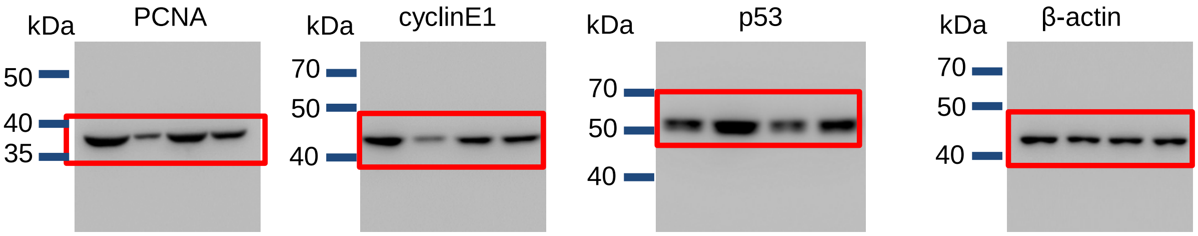

Supplementary Fig. 7a

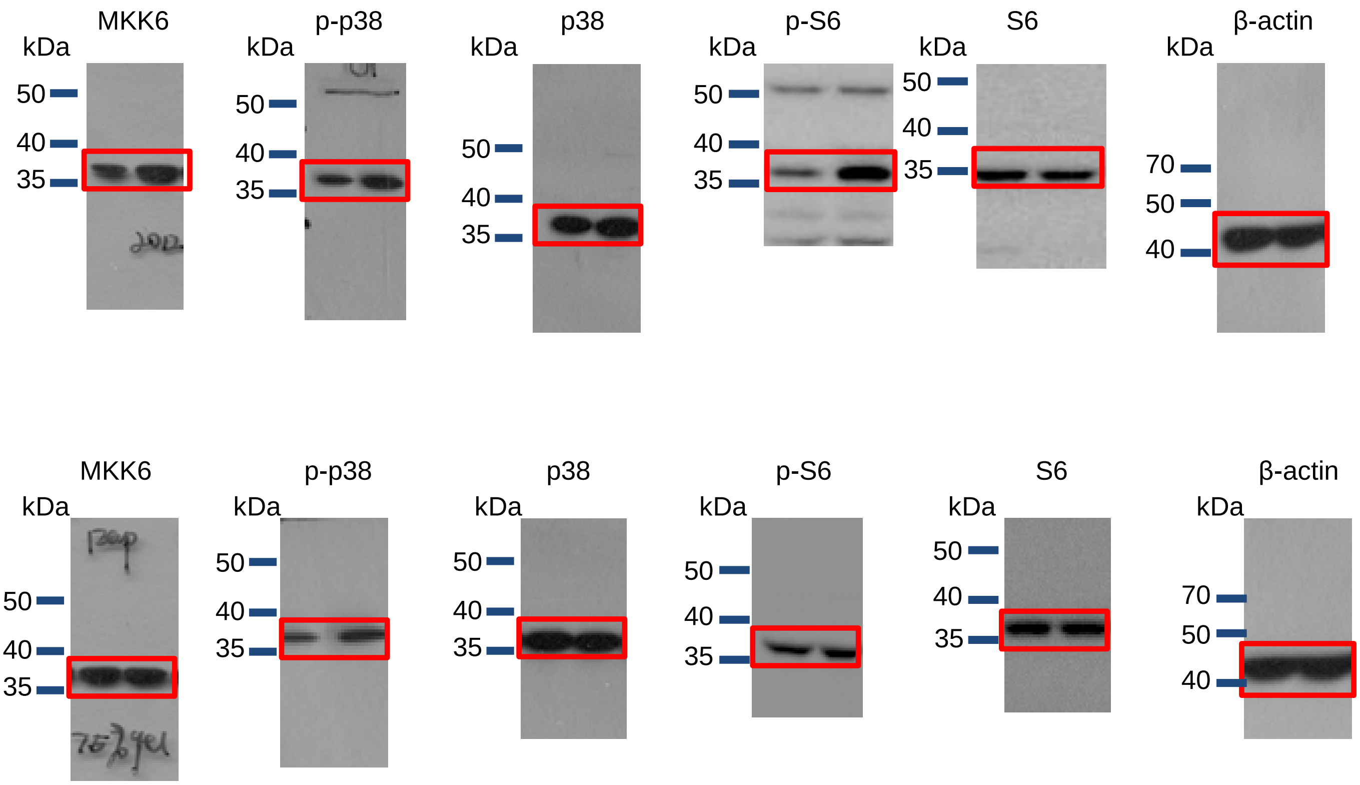

Supplementary Fig. 7c

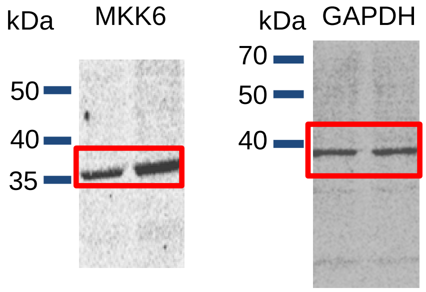

Supplementary Fig. 7d

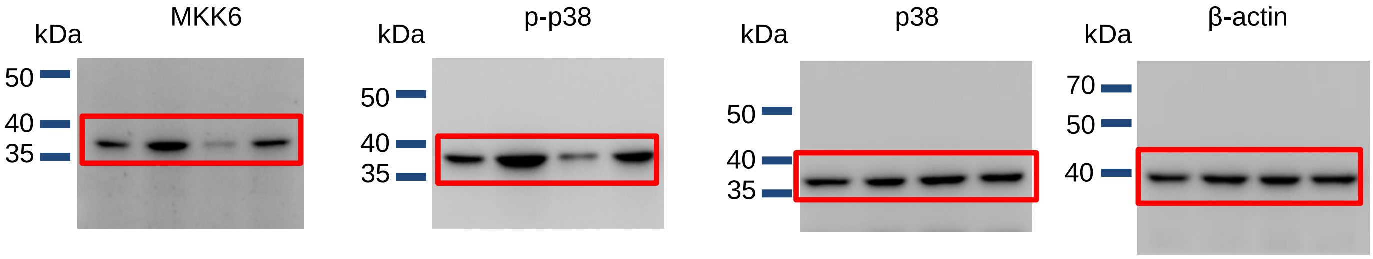

Supplementary Fig. 8a

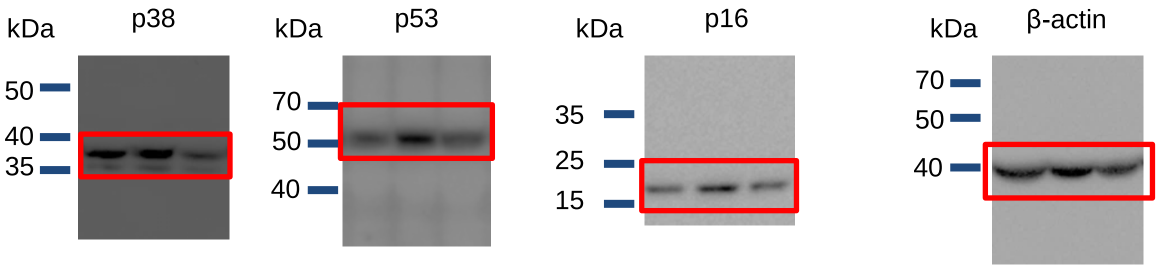

Supplementary Fig. 9a

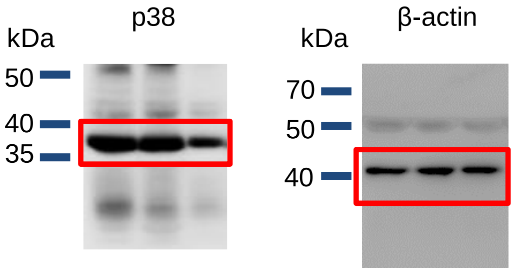

Supplementary Fig. 9d

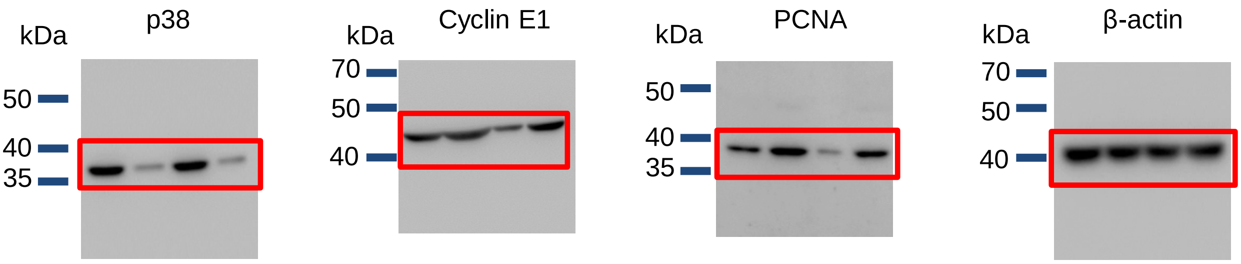

Fig. s9f.

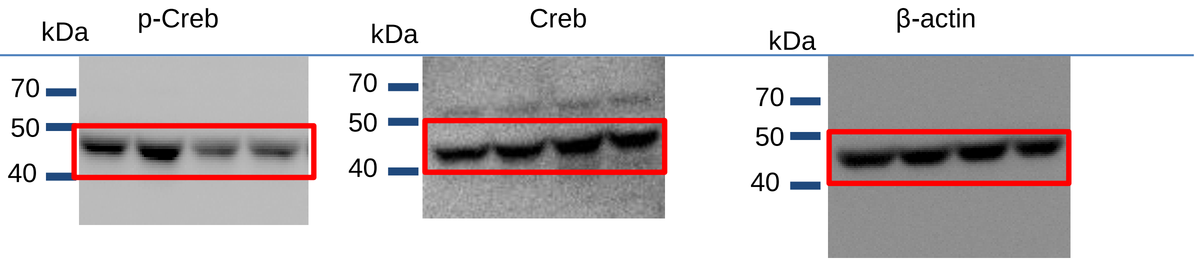

Supplementary Fig. 10a

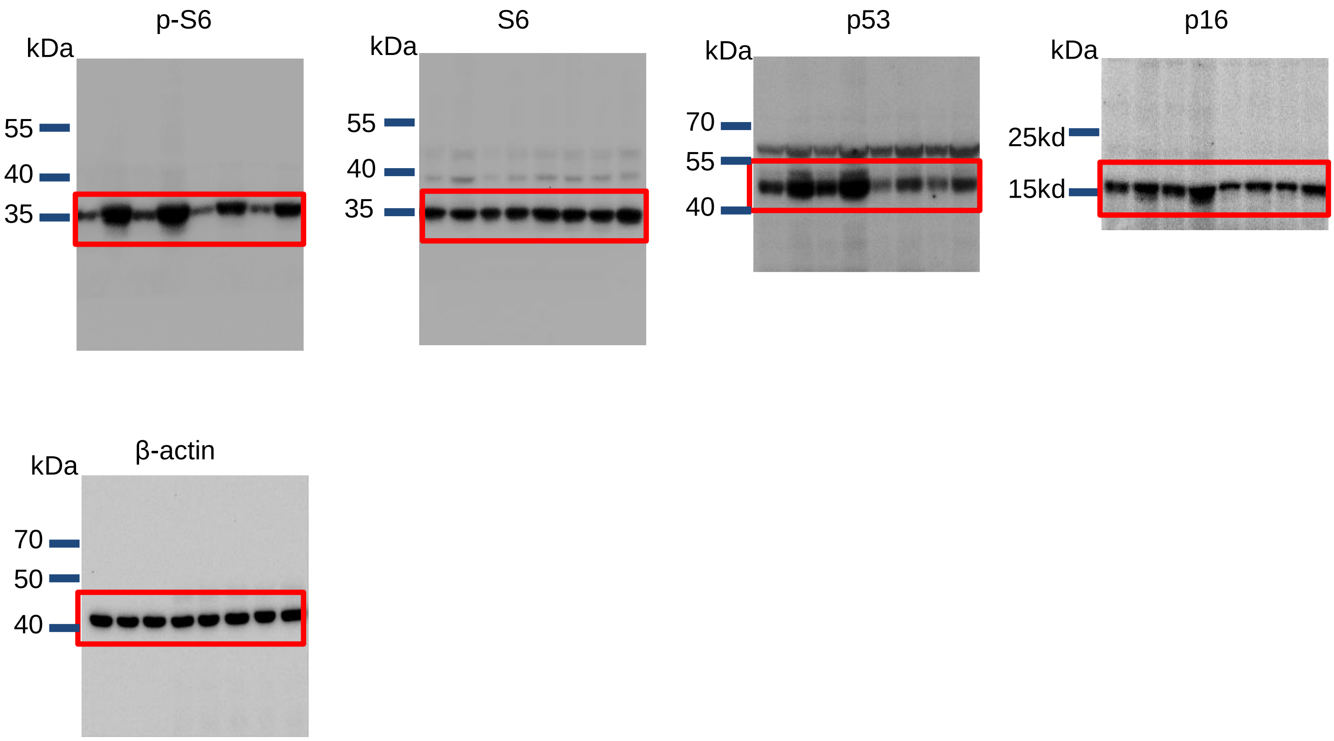

Supplementary Fig. 10b

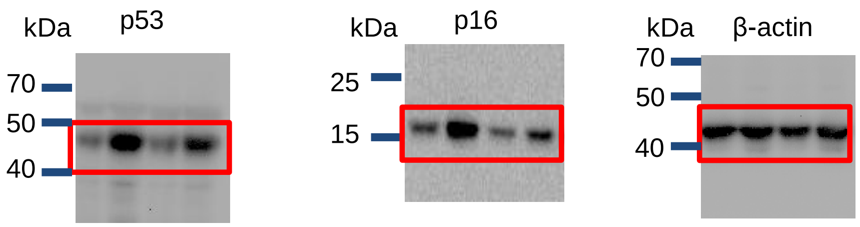

Fig. s10c.

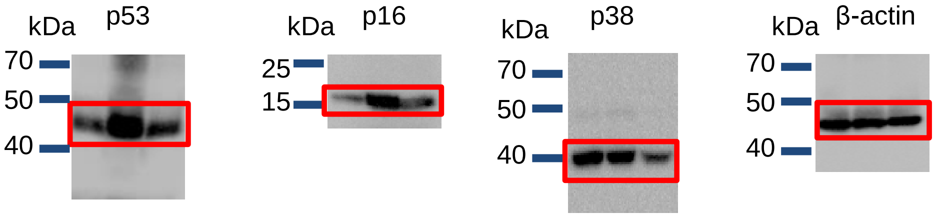

Supplementary Table 1

Protein List	Signals (WT)	Signals (KO)	KO/WT	
ATRIP (Ab-68/72)	676.50	2344.33	3.47	
Bax (N-term)	351.00	553.83	1.58	
BCL-2 (Phospho-Ser87)	133.33	212.33	1.59	
Beta actin	168.50	280.00	1.66	
BRCA1 (Ab-1423)	3750.50	10391.67	2.77	
Caspase-3 (Phospho-Ser150)	137.60	213.67	1.55	
CDK2 (Ab-160)	136.00	245.67	1.81	
Chk1 (Ab-286)	180.83	420.33	2.32	
Chk1 (Ab-317)	128.83	296.17	2.30	
Chk2 (Ab-383)	266.67	406.33	1.52	
Chk2 (Ab-68)	369.33	2164.50	5.86	
Chk2 (Phospho-Ser516)	144.83	228.83	1.58	
Cyclin D1 (ab-286)	837.00	1610.60	1.92	
Cyclin D1 (Ab-90)	219.33	374.83	1.71	
Cyclin D1 (Phospho-Thr286)	237.17	735.00	3.10	
Cyclin E1 (Ab-77)	1691.40	3589.40	2.12	
DNA-PK (Ab-2638)	178.83	370.83	2.07	
E2F1 (Ab-433)	323.50	1301.17	4.02	
ERK3 (Phospho-Ser189)	125.20	191.17	1.53	
HDAC1 (Ab-421)	5833.50	11575.67	1.98	
HDAC1 (Phospho-Ser421)	187.40	309.33	1.65	
HDAC2 (Phospho-Ser394)	196.50	363.33	1.85	
HDAC3 (Ab-424)	261.33	703.33	2.69	
HDAC4 (Phospho-Ser632)	158.00	263.67	1.67	
HDAC5 (Phospho-Ser259)	154.50	260.00	1.68	
HDAC5 (Phospho-Ser498)	117.80	188.83	1.60	
MAPKAPK2 (Ab-272)	125.50	190.67	1.52	
MAPKAPK2 (Phospho-Ser272)	186.67	424.00	2.27	
MDM2 (Ab-166)	4277.83	11611.00	2.71	
MEK1 (Ab286)	505.33	1800.67	3.56	
MEK1 (Ab-291)	167.20	375.83	2.25	
MEK1 (Ab-298)	573.17	2611.50	4.56	
p38 MAPK (Ab-182)	576.00	4153.17	7.21	
p53 (Ab-18)	161.40	420.00	2.60	
p53 (Ab-20)	839.60	2073.50	2.47	
p53 (Ab-33)	184.67	379.20	2.05	
p53 (Ab-37)	166.00	266.83	1.61	
p53 (Ab-392)	202.17	303.50	1.50	
Positive Marker	8036.20	12036.60	1.50	
Raf1 (Ab-341)	138.83	219.50	1.58	
Rb (Ab-780)	177.40	382.00	2.15	
Rb (Ab-795)	162.17	485.67	2.99	
Rb (Ab-807)	207.00	545.60	2.64	
RBAK (inter)	133.40	625.33	4.69	
RBM5 (inter)	162.00	2247.20	13.87	

Supplementary Table 1. Increased expression and activation of molecules involved in proliferation in intestine samples. Proteins with KO/WT ratios greater than 1.5 were shown.
