# Supplementary material for: Gut stem cell aging is driven by mTORC1 via a p38 MAPK-p53 pathway
Source: Nat Commun. 2020 Jan 2;11:37. doi: 10.1038/s41467-019-13911-x (PMC6940394; doi:10.1038/s41467-019-13911-x)
Supplement: Supplementary file 3 — Reporting Summary [file 41467_2019_13911_MOESM3_ESM.pdf]

nature research

Corresponding author(s): Baojie Li

Last updated by author(s): Nov 15, 2019

Reporting Summary

Nature Research wishes to improve the reproducibility of the work that we publish. This form provides structure for consistency and transparency in reporting. For further information on Nature Research policies, see Authors & Referees and the Editorial Policy Checklist.

Statistics

For all statistical analyses, confirm that the following items are present in the figure legend, table legend, main text, or Methods section.

n/a	Confirmed	
☐	☒	The exact sample size (n) for each experimental group/condition, given as a discrete number and unit of measurement
☐	☒	A statement on whether measurements were taken from distinct samples or whether the same sample was measured repeatedly
☐	☒	The statistical test(s) used AND whether they are one- or two-sided<br>*Only common tests should be described solely by name; describe more complex techniques in the Methods section.*
☒	☐	A description of all covariates tested
☒	☐	A description of any assumptions or corrections, such as tests of normality and adjustment for multiple comparisons
☒	☐	A full description of the statistical parameters including central tendency (e.g. means) or other basic estimates (e.g. regression coefficient) AND variation (e.g. standard deviation) or associated estimates of uncertainty (e.g. confidence intervals)
☒	☐	For null hypothesis testing, the test statistic (e.g. F, t, r) with confidence intervals, effect sizes, degrees of freedom and P value noted<br>*Give P values as exact values whenever suitable.*
☒	☐	For Bayesian analysis, information on the choice of priors and Markov chain Monte Carlo settings
☒	☐	For hierarchical and complex designs, identification of the appropriate level for tests and full reporting of outcomes
☒	☐	Estimates of effect sizes (e.g. Cohen's d, Pearson's r), indicating how they were calculated

Our web collection on statistics for biologists contains articles on many of the points above.

Software and code

Policy information about availability of computer code

Data collection	Data in this manuscript are predominantly microscopy data. These data were collected using commercial software that are associated with the microscopes used (see Materials and Methods).
Data analysis	For data analysis, we used Graphpad which is available at https://www.graphpad.com.

For manuscripts utilizing custom algorithms or software that are central to the research but not yet described in published literature, software must be made available to editors/reviewers. We strongly encourage code deposition in a community repository (e.g. GitHub). See the Nature Research guidelines for submitting code & software for further information.

Data

Policy information about availability of data

All manuscripts must include a data availability statement. This statement should provide the following information, where applicable:

- Accession codes, unique identifiers, or web links for publicly available datasets
- A list of figures that have associated raw data
- A description of any restrictions on data availability

a full data availability statement has been included in the mansuript.

Field-specific reporting

Please select the one below that is the best fit for your research. If you are not sure, read the appropriate sections before making your selection.

☒ Life sciences ☐ Behavioural & social sciences ☐ Ecological, evolutionary & environmental sciences

Life sciences study design

All studies must disclose on these points even when the disclosure is negative.

Sample size	We did not predetermined the sample size.The number of samples/experiments for each data panel has been described in the figure legends.
Data exclusions	No data were excluded from the analyses.
Replication	We carried out our experiments at least three times or used at least three animals in each group.
Randomization	Experimental groups were defined by experimental conditions. Within an experimental condition, the allocation was random.
Blinding	All experimental procedures and quantification of results including drug injections and tissue histological analysis were done by two independent researchers.

Reporting for specific materials, systems and methods

We require information from authors about some types of materials, experimental systems and methods used in many studies. Here, indicate whether each material, system or method listed is relevant to your study. If you are not sure if a list item applies to your research, read the appropriate section before selecting a response.

Materials & experimental systems

n/a	Involved in the study	
☐	☒	Antibodies
☒	☐	Eukaryotic cell lines
☒	☐	Palaeontology
☐	☒	Animals and other organisms
☒	☐	Human research participants
☒	☐	Clinical data

Methods

n/a	Involved in the study	
☒	☐	ChIP-seq
☒	☐	Flow cytometry
☒	☐	MRI-based neuroimaging

Antibodies

Antibodies used	All the antibodies used in the mansuript have a full describtion in the online methods
Validation	All the antibodies are validated and provided on the manufacturer's website

Animals and other organisms

Policy information about studies involving animals; ARRIVE guidelines recommended for reporting animal research

Laboratory animals	Male C57B6 mice (2-17.5 month,18-26 g) were purchased from Charles River Laboratories China. The genetically modified animals are on B6 background.
Wild animals	The study did not involve wild animals.
Field-collected samples	This study did not involve samples collected from the field.
Ethics oversight	Mouse experiments were performed in accordance with the Guide for the Care and Use of Laboratory Animals and approved by the Institution Animal Care and Use Committee (IACUC) of Shanghai Jiao Tong University.

Note that full information on the approval of the study protocol must also be provided in the manuscript.
