# Supplementary material for: Gut stem cell aging is driven by mTORC1 via a p38 MAPK-p53 pathway
Source: Nat Commun. 2020 Jan 2;11:37. doi: 10.1038/s41467-019-13911-x (PMC6940394; doi:10.1038/s41467-019-13911-x)
Supplement: Supplementary file 2 — Peer Review File [file 41467_2019_13911_MOESM2_ESM.pdf]

Reviewers' comments:

Reviewer #1 (Remarks to the Author):

In this study the authors report a novel role for MTOR signalling in regulating ageing and tissue degradation in the intestine. Using a number of genetic models the authors propose that MTOR activation in ageing intestine leads to an activation of p38 MAPK and p53 signalling leading to depletion of intestinal progenitor cell proliferation and subsequent degradation of villus function.

This is an interesting study and the major claims are backed by the data presented. The data appears robust and the genetic models used support a role for MTOR activation in regulating p38 activation and this in turn being responsible for at least some of the aspects of tissue degradation observed. The work presented convincingly supports to authors conclusions and I recommend it is accepted for publication with a number of minor corrections:

1) The authors propose MTOR activation in old mice is the key mediator of intestinal ageing but the evidenced presented in Fig 1e and f needs to be strengthened with additional members of the MTOR signalling pathway (pMTOR, p4EBP1) analysed. Additionally, quantification of Fig 1f should be included.
2) Could the authors report what happens to normal ISCs in old mice? They find reductions in ISC numbers in aged Tscfl/fl mice (Fig 3e). Are ISC numbers also reduced in naturally aged mice?
3) I thought the language used to describe some of the experiments was a bit strong and not completely supported by the evidence provided. For example, on page 6 (line 116) the authors state the Tsc1 KO experiment will 'prove' the role of MTORC1. I think this is overstating the evidence it will provide as Tsc1 also has other reported roles, such as protein chaperone function. Therefore, it is likely the depletion of Tsc1 will have additional effects out with MTORC1. I would tone down the language used when describing the mechanistic link in this section. The p53 KO experiments in figure 7 should come with the same caveats due to the huge number of functions regulated by p53.

Reviewer #2 (Remarks to the Author):

This is an interesting and very comprehensive paper that shows that hyperactive mTORC1 (by Tsc1 loss) can produce phenotypes that resemble the ones associated with extreme aging. Even though Tsc1 ablation produces intestinal cell overgrowth in young mice as formerly reported, it depletes stem cell and progenitor cell population during aging and causes premature aging phenotype in the intestine. As the mechanism, the authors report that mTORC1 can specifically upregulate MKK6 translation in intestinal cells, leading to p38 activation that depletes the intestinal stem cells. Treatment with p38 inhibitors, or instestinal stem cell-specific p38 ablation, attenuated intestinal aging like rapamycin. These are interesting and important addition to the field. Following comments are for consideration of the authors to further improve their manuscript.

1. Fig. 1c-d: Ki67 staining is not so convincing, and the section staining quality might be quite different between the samples. Re-analyzing the samples in western blot (similar to Fig. 1f) for cell proliferation markers could be helpful. It would be much more convincing if proliferation markers decrease while mTORC1 activity markers are increased in the same samples. Similar assays could be done for the other types of experiments (e.g. Tsc1 knockout animals) to more objectively quantify the effects on the proliferative capacities of the intestine.

2. Tsc1 and p38 are intestine-specifically modulated. But mTORC1 was not modulated in such a way. Although aging effect and Tsc1 effect is similar in some aspects, it is not clear whether pathogenetic mechanisms are the same. It is actually expected that Tsc1 ablation will cause some stem cell

pathologies, which can be even unrelated to aging, due to the mTORC1 hyperactivation. Therefore, it would be more important to show whether mTORC1 inhibition can attenuate the normal gut aging. But, in the authors' data, rapamycin was systematically administered, and this could be problematic because systematic rapamycin administration was already known to attenuate aging in multiple tissues and readouts. So, it is unclear whether rapamycin effect is indeed specific for intestines and intestinal stem cells or through indirect mechanisms. To resolve this, the authors should test mTOR- or Raptor-floxed mice and delete (or inhibit) mTORC1 specifically in stem cells. Alternatively, rapamycin should be treated to isolated organoids to see if the effect of aging and Tsc1 ablation can be indeed reversed by such treatments (see my comment below in 3).

3. Enteroids from 2 and 8 m old WT and Tsc1-knockout mice were analyzed in Fig. 3. Could the authors analyze 17.5 m old WT mice and see if they show similar pathologies like Tsc1-knockout enteroids? If the aging effects are the same as Tsc1 ablation effects, this could be replicated. In addition, can Tsc1 defects and 17.5 m old aging defects (if similar to Tsc1 defects) be restored by rapamycin treatments or p38 inhibition in these models? This is an isolated enteroid system, so the results would be more specific to the intestinal system (unlike systemic anti-aging effects of rapamycin).

4. It is a very interesting observation that MKK6 protein translation is controlled by mTORC1. However, data were not provided to support if this protein increase is indeed the cause of p38 activation by mTORC1. It is more likely that, in addition to increased MKK6 expression, mTORC1-induced proteostatic stress or oxidative stress contributes to the activation of p38 MAPK. In this context, the linear model in Fig. 7f could be an oversimplified one. For instance, it is most likely that increased mTORC1 produces additional ER stress or oxidative damage to activate p38 MAPK.

Reviewer #3 (Remarks to the Author):

This is a very interesting study that suggests continuous activation of mTORC1 in crypt base columnar intestinal stem cells drives ageing phenotypes in the intestinal epithelium through activation of a p38-p53 pathway. This paper is definitely a strong candidate for publication, however I have a couple of concerns, starting with the most major, involving the radiation experiments. The suggestion that mTORC1 inhibition using Rapamycin somehow enhances regeneration of the intestinal epithelium, both in young and aged (3.5 and 17.5 month) mice, is in stark contrast with numerous published studies from several groups in different locations (e.g., Ashton GH Dev Cell 2010, Sampson LL et al FASEB J March 2016, Guan Y et al. J. Immunology July 2015, Yousefi M et al. Stem Cell Reports March 2018, etc.).

That the authors find the complete opposite effect observed in these numerous studies from multiple independent groups is a bit disconcerting. In the current study, the authors present data from 6 days after radiation injury, which is unusual since in healthy mice (at least in non-aged healthy mice), the epithelium is fully regenerated after this long, even after much higher doses of injury. Further, the histological evidence presented in the study show only high magnification images of a few crypts for each condition.

Ultimately, for the rest of this study to be taken at face value, the authors should reconcile this obvious discrepancy, at the very least by showing low magnification images encompassing many crypt-villus axes, ideally stained for a proliferative marker or BrdU-pulsed, at more relevant timepoints- 48, 72 hours post-irradiation.

Beyond this major concern, there is another point of interest regarding the interpretation of the data.

To this reviewer, the most likely explanation for the phenotypes observed is that mTORC1 activation eventually drives stem cell exhaustion. This could be examined in the Lgr5-Cre-LSL-tomato mice, in the presence or absence of TSC floxed alleles, using lower, clonal doses of Tam. The prediction would be that at early timepoints after Cre activation, TSC wt and knockout mice would exhibit similar numbers of tracing events, while at longer timepoints, stem cell exhaustion would result in a loss of tracing events in the TSC knockouts relative to wildtypes.

Such a finding would be consistent with the authors' observations in 3a, showing TSC ablation results in lower CBC numbers in the absence of obvious increases in apoptosis. A stem cell exhaustion interpretation is also totally consistent with the organoid assay in 3d- transient increases in stem cell activity upon TSC deletion followed by a crash due to exhaustion, as well as the in vivo correlate in 3e.

An interpretation of stem cell exhaustion seems much more logical that the purported 'biphasic role' for mTORC1 in growth and ageing. I'm curious whether the authors consider this interpretation, and if they reject this interpretation, why?

Additional, more minor issues:

Type of stat tests used should be included in figure legends

Figure 1e, 1g, S1j, and figures related to mTORC activity: mTORC activity is highly sensitive to feeding- how was this controlled for when sacrificing animals? i.e., where the mice fasted overnight to insure that the apparent changes in mTORC activity were not simply a result of feeding (and altered feeding patterns with age)?

4d- protein synthesis rates were not examined, and thus concluding translation rates are change is a bit speculative, as it could be stability.

Repeated referral to antibody arrays, but the data is not shown?

Reviewers' comments:

Reviewer #1 (Remarks to the Author):

In this study the authors report a novel role for MTOR signalling in regulating ageing and tissue degradation in the intestine. Using a number of genetic models the authors propose that MTOR activation in ageing intestine leads to an activation of p38 MAPK and p53 signalling leading to depletion of intestinal progenitor cell proliferation and subsequent degradation of villus function.
This is an interesting study and the major claims are backed by the data presented. The data appears robust and the genetic models used support a role for MTOR activation in regulating p38 activation and this in turn being responsible for at least some of the aspects of tissue degradation observed. The work presented convincingly supports to authors conclusions and I recommend it is accepted for publication with a number of minor corrections:
We appreciate the reviewer’s helpful comments and valuable suggestions.

1) The authors propose MTOR activation in old mice is the key mediator of intestinal ageing but the evidenced presented in Fig 1e and f needs to be strengthened with additional members of the MTOR signalling pathway (pMTOR, p4EBP1) analysed. Additionally, quantification of Fig 1f should be included.
We have now included WB results for p-mTOR, pS6K, and p-4EBP1, IHC results for p-4EBP1 (Fig. 1f and 1g), and the quantitation data including that for original Fig. 1f in the revised manuscript.

2) Could the authors report what happens to normal ISCs in old mice? They find reductions in ISC numbers in aged Tscfl/fl mice (Fig 3e). Are ISC numbers also reduced in naturally aged mice?
We analyzed the intestinal villi of 2 and 17.5 month-old Lgr5-GFP-CreERT mice and found that the numbers of Lgr5+ ISCs and proliferating ISCs were decreased (new Fig. 3f).

3) I thought the language used to describe some of the experiments was a bit strong and not completely supported by the evidence provided. For example, on page 6 (line

116) the authors state the Tsc1 KO experiment will 'prove' the role of MTORC1. I think this is overstating the evidence it will provide as Tsc1 also has other reported roles, such as protein chaperone function. Therefore, it is likely the depletion of Tsc1 will have additional effects out with MTORC1. I would tone down the language used when describing the mechanistic link in this section. The p53 KO experiments in figure 7 should come with the same caveats due to the huge number of functions regulated by p53.
We have rewritten these parts of the text to tone down the claims regarding the mechanisms.

Reviewer #2 (Remarks to the Author):

This is an interesting and very comprehensive paper that shows that hyperactive mTORC1 (by Tsc1 loss) can produce phenotypes that resemble the ones associated with extreme aging. Even though Tsc1 ablation produces intestinal cell overgrowth in young mice as formerly reported, it depletes stem cell and progenitor cell population during aging and causes premature aging phenotype in the intestine. As the mechanism, the authors report that mTORC1 can specifically upregulate MKK6 translation in intestinal cells, leading to p38 activation that depletes the intestinal stem cells. Treatment with p38 inhibitors, or intestinal stem cell-specific p38 ablation, attenuated intestinal aging like rapamycin. These are interesting and important addition to the field. Following comments are for consideration of the authors to further improve their manuscript.
We appreciate the reviewer's helpful comments and valuable suggestions.

1. Fig. 1c-d: Ki67 staining is not so convincing, and the section staining quality might be quite different between the samples. Re-analyzing the samples in western blot (similar to Fig. 1f) for cell proliferation markers could be helpful. It would be much more convincing if proliferation markers decrease while mTORC1 activity markers are increased in the same samples. Similar assays could be done for the other types of experiments (e.g. Tsc1 knockout animals) to more objectively quantify the effects on the proliferative capacities of the intestine.
We have now done WB analysis for PCNA, cyclin E and p53 using the intestinal samples (Fig. S2b), and found that pro-proliferation markers were down and anti-proliferation marker was up in old mice, which were rescued by RAP. We have done similar assays with *Lgr5-CreERT; Tsc1*$^{f/f}$ mice and *Lgr5-CreERT; Tsc1*$^{f/f}$*; Mapk14*$^{f/f}$ mice (Fig. S5c and S9d), and the results support our conclusions. These results are now included in the revised manuscript.

2. Tsc1 and p38 are intestine-specifically modulated. But mTORC1 was not modulated in such a way. Although aging effect and Tsc1 effect is similar in some aspects, it is not clear whether pathogenetic mechanisms are the same. It is actually expected that Tsc1 ablation will cause some stem cell pathologies, which can be even unrelated to aging, due to the mTORC1 hyperactivation. Therefore, it would be more

important to show whether mTORC1 inhibition can attenuate the normal gut aging. But, in the authors' data, rapamycin was systematically administered, and this could be problematic because systematic rapamycin administration was already known to attenuate aging in multiple tissues and readouts. So, it is unclear whether rapamycin effect is indeed specific for intestines and intestinal stem cells or through indirect mechanisms. To resolve this, the authors should test mTOR- or Raptor-floxed mice and delete (or inhibit) mTORC1 specifically in stem cells. Alternatively, rapamycin should be treated to isolated organoids to see if the effect of aging and Tsc1 ablation can be indeed reversed by such treatments (see my comment below in 3).
We have tried the *Lgr5-CreERT;mTor*$^{f/f}$ mice. However, these mice showed smaller villi and deceased cell proliferation, confirming that mTOR is absolutely required for ISC turnover and that the mice are not suitable for aging-related studies. So we used organoid cultures to test this point. We cultured organoids from 2- and 17.5-month-old WT mice and added RAP (0.5 μM). We found that RAP was able to partially rescue the aging-like phenotypes (organoid size and crypt formation), suggesting that the effect of RAP is directly on ISCs/TA cells. We have included these results and the quantitation data in the revised manuscript (Fig. 6e).

3. Enteroids from 2 and 8 m old WT and Tsc1-knockout mice were analyzed in Fig. 3. Could the authors analyze 17.5 m old WT mice and see if they show similar pathologies like Tsc1-knockout enteroids? If the aging effects are the same as Tsc1 ablation effects, this could be replicated. In addition, can Tsc1 defects and 17.5 m old aging defects (if similar to Tsc1 defects) be restored by rapamycin treatments or p38 inhibition in these models? This is an isolated enteroid system, so the results would be more specific to the intestinal system (unlike systemic anti-aging effects of rapamycin).
We carried out organoid cultures using 2 and 17.5-month-old mice and found that naturally-aged mice did have reduced numbers of ISCs. We then cultured organoids from 2- and 17.5-month-old WT mice in the presence of RAP (0.5 μM) or SB203580 (2 μM). We found that RAP or SB203580 was able to partially rescue the aging phenotypes, suggesting that their effects are directly on ISCs/TA cells. We have included these results and the quantitation data in the revised manuscript (Fig. 6e).

4. It is a very interesting observation that MKK6 protein translation is controlled by mTORC1. However, data were not provided to support if this protein increase is indeed the cause of p38 activation by mTORC1. It is more likely that, in addition to increased MKK6 expression, mTORC1-induced proteostatic stress or oxidative stress contributes to the activation of p38 MAPK. In this context, the linear model in Fig. 7f could be an oversimplified one. For instance, it is most likely that increased mTORC1 produces additional ER stress or oxidative damage to activate p38 MAPK.
We knocked down MKK6 in Tsc1$^{-/-}$ cells and found that this led a decrease in p38MAPK activation (new Fig. S7d). In addition, we have rewritten this part of text to include the possible contribution of ER/proteostatic stress and oxidative stress to activation of p38MAPKs and modified the model accordingly.

Reviewer #3 (Remarks to the Author):

This is a very interesting study that suggests continuous activation of mTORC1 in crypt base columnar intestinal stem cells drives ageing phenotypes in the intestinal epithelium through activation of a p38-p53 pathway. This paper is definitely a strong candidate for publication, however I have a couple of concerns, starting with the most major, involving the radiation experiments. The suggestion that mTORC1 inhibition using Rapamycin somehow enhances regeneration of the intestinal epithelium, both in young and aged (3.5 and 17.5 month) mice, is in stark contrast with numerous published studies from several groups in different locations (e.g., Ashton GH Dev Cell 2010, Sampson LL et al FASEB J March 2016, Guan Y et al. J. Immunology July 2015, Yousefi M et al. Stem Cell Reports March 2018, etc.).
We appreciate the reviewer's helpful comments and valuable suggestions

That the authors find the complete opposite effect observed in these numerous studies from multiple independent groups is a bit disconcerting. In the current study, the authors present data from 6 days after radiation injury, which is unusual since in healthy mice (at least in non-aged healthy mice), the epithelium is fully regenerated after this long, even after much higher doses of injury. Further, the histological evidence presented in the study show only high magnification images of a few crypts for each condition.
Ultimately, for the rest of this study to be taken at face value, the authors should reconcile this obvious discrepancy, at the very least by showing low magnification images encompassing many crypt-villus axes, ideally stained for a proliferative marker or BrdU-pulsed, at more relevant time points- 48, 72 hours post-irradiation.
We agree that this is a very important issue. It is well established that mTOR signaling is required for cell proliferation during growth and regeneration of various tissues including the intestine villi and ISCs (we had cited those papers in the original submission). Indeed, we found that in *Lgr5-CreERT;mTor*$^{f/f}$ mice, TAM administration led to quick decrease in villus size and TA cell proliferation (data not shown). Following the suggestions of the reviewer, we have repeated the experiments and harvested the samples 48, 72 hours post-irradiation (5 Gy). We found that mTOR hyperactivation (by Tsc1 deletion or during to aging) rendered ISC/TA cells increased sensitivity to IR-induced decreases in the numbers of crypts and cell proliferation and increase in apoptosis at day 2 or 3 post IR (see new data for 17.5-month-old mice, Tsc1 deficient mice, Tsc1 and Makp14 double KO mice at day 2 or 3 post IR). The more severe damage may directly cause regeneration problems. Moreover, we found that RAP was able to rescue the increased sensitivity to IR including decreases in the numbers of crypts and cell proliferation and increase in apoptosis at day 2 or 3 post IR. This will certainly help the regeneration process.
We included all these new data (low magnification images) and clarified this issue in the revised manuscript. See new Fig. 1c, 2d, S2c, S5d, S9b.

Beyond this major concern, there is another point of interest regarding the interpretation of the data. To this reviewer, the most likely explanation for the phenotypes observed is that mTORC1 activation eventually drives stem cell exhaustion. This could be examined in the Lgr5-Cre-LSL-tomato mice, in the presence or absence of TSC floxed alleles, using lower, clonal doses of Tam. The prediction would be that at early timepoints after Cre activation, TSC wt and knockout mice would exhibit similar numbers of tracing events, while at longer time points, stem cell exhaustion would result in a loss of tracing events in the TSC knockouts relative to wildtypes.

The reviewer suggested to use Lgr5-CreERT; Rosa-LSL-Tomato; Tsc1f/f mice and Lgr5-CreERT; Rosa-LSL-Tomato mice. TAM injection will lead to simultaneous Tomato labelling and Tsc1 deletion. We had presented the tracing results for Lgr5-CreERT; Rosa-LSL-Tomato mice (see supplementary Fig. S4a), 65% of crypts and villi were Tomato+ 4 weeks after TAM administration. The prediction will be that a long-term deletion of Tsc1 would lead to fewer Tomato+ crypts and villi. These mice can be used to determine the effect of Tsc1 deficiency on the activity of Lgr5+ ISCs, which drive fast villus turnover (4-5 days per cycle). While these mice can be useful, they will take a very long time to get (3 transgenes in one mouse). Fortunately, the Lgr5-EGF-CreERT mice used in this study carries GFP driven by Lgr5 promoter. We could easily determine the numbers of active ISCs by GFP fluorescence and their activity by co-staining for PCNA or Ki67. Moreover, we can directly count the numbers of villi and crypts and measure the size of them in these mice. Based on this, we think that it is not necessary to include these triple transgenic mouse data in this manuscript. A potential problem with low dose of TAM is that it may delete Tsc1 and activate Tomato in different cells.

Such a finding would be consistent with the authors' observations in 3a, showing TSC ablation results in lower CBC numbers in the absence of obvious increases in apoptosis. A stem cell exhaustion interpretation is also totally consistent with the organoid assay in 3d- transient increases in stem cell activity upon TSC deletion followed by a crash due to exhaustion, as well as the in vivo correlate in 3e.

An interpretation of stem cell exhaustion seems much more logical that the purported 'biphasic role' for mTORC1 in growth and ageing. I'm curious whether the authors consider this interpretation, and if they reject this interpretation, why?

The decrease in the number of CBCs can be explained by stem cell exhaustion. The point we wanted to make is that stem cell exhaustion is caused by enhanced stress response but not by replicative senescence. We have discussed this in the revised manuscript.

Additional, more minor issues:

Type of stat tests used should be included in figure legends

We have detailed the statistical methods.

Figure 1e, 1g, S1j, and figures related to motors activity: motors activity is highly sensitive to feeding- how was this controlled for when sacrificing animals? i.e., where the mice fasted overnight to insure that the apparent changes in motors activity were not simply a result of feeding (and altered feeding patterns with age)?
In these experiments, the mice were starved overnight and harvested in the following morning. We have included this description in Materials and Methods.

4d- protein synthesis rates were not examined, and thus concluding translation rates are change is a bit speculative, as it could be stability.
We agree with the reviewer. We had done stability assays long time ago and found that MKK6 was rather stable and its stability was not affected by Tsc1 deficiency. We have included this information in the revised manuscript and rewritten this part of text. If requested, we can include these results in the supplementary information.

Repeated referral to antibody arrays, but the data is not shown?
The assay was done by a commercial service and the results were summarized in Table S1.

REVIEWERS' COMMENTS:

Reviewer #1 (Remarks to the Author):

The authors have addressed my comments and I now recommend publication.

Kevin Myant

Reviewer #2 (Remarks to the Author):

The authors responded well to my former critiques. By performing additional experiments requested by me and the other reviewers, the paper was substantially improved.

Reviewer #3 (Remarks to the Author):

The authors have addressed most of my concerns, but my major concerns remains somewhat unaddressed. In my prior review, the statement was as follows:

"This is a very interesting study that suggests continuous activation of mTORC1 in crypt base columnar intestinal stem cells drives ageing phenotypes in the intestinal epithelium through activation of a p38-p53 pathway. This paper is definitely a strong candidate for publication, however I have a couple of concerns, starting with the most major, involving the radiation experiments. The suggestion that mTORC1 inhibition using Rapamycin somehow enhances regeneration of the intestinal epithelium, both in young and aged (3.5 and 17.5 month) mice, is in stark contrast with numerous published studies from several groups in different locations (e.g., Ashton GH Dev Cell 2010, Sampson LL et al FASEB J March 2016, Guan Y et al. J. Immunology July 2015, Yousefi M et al. Stem Cell Reports March 2018, etc.).

That the authors find the complete opposite effect observed in these numerous studies from multiple independent groups is a bit disconcerting. In the current study, the authors present data from 6 days after radiation injury, which is unusual since in healthy mice (at least in non-aged healthy mice), the epithelium is fully regenerated after this long, even after much higher doses of injury. Further, the histological evidence presented in the study show only high magnification images of a few crypts for each condition.
Ultimately, for the rest of this study to be taken at face value, the authors should reconcile this obvious discrepancy, at the very least by showing low magnification images encompassing many crypt-villus axes, ideally stained for a proliferative marker or BrdU-pulsed, at more relevant time points- 48, 72 hours post-irradiation"

The authors respond:

"We agree that this is a very important issue. It is well established that mTOR signaling is required for cell proliferation during growth and regeneration of various tissues including the intestine villi and ISCs (we had cited those papers in the original submission). Indeed, we found that in Lgr5-CreERT;mTorf/f mice, TAM administration led to quick decrease in villus size and TA cell proliferation (data not shown). Following the suggestions of the reviewer, we have repeated the experiments and harvested the samples 48, 72 hours post-irradiation (5 Gy). We found that mTOR hyperactivation (by Tsc1 deletion or during to aging) rendered ISC/TA cells increased sensitivity to IR-induced decreases in the numbers of crypts and cell proliferation and increase in apoptosis at day 2 or 3 post IR (see new

data for 17.5-month-old mice, Tsc1 deficient mice, Tsc1 and Makp14 double KO mice at day 2 or 3 post IR). The more severe damage may directly cause regeneration problems. Moreover, we found that RAP was able to rescue the increased sensitivity to IR including decreases in the numbers of crypts and cell proliferation and increase in apoptosis at day 2 or 3 post IR. This will certainly help the regeneration process.
We included all these new data (low magnification images) and clarified this issue in the revised manuscript. See new Fig. 1c, 2d, S2c, S5d, S9b"

So I commend the authors for doing these experiments, yet the new data reveal that the central concern remains: young mice treated with rapamycin then irradiated show no difference in the number of regenerative crypt foci in new Figure 1c, and old mice actually show an increase. Again, this is in stark contrast (at least with respect to the young mice) to the current literature (some cited in my original comment), that Rap treatment has been shown by numerous labs to be a potent inhibitor post-IR crypt regeneration. The authors didn't address this discrepancy between their findings, and the opposing findings from these other labs in the literature.

I still think that this stark discrepancy needs to be addressed. What could account for this study observing results opposite to what's in the literature? How do the doses of Rap and radiation and/or timing of treatment and analysis compare between their study and those in the literature? How could one possibly account for this discrepancy?

REVIEWERS' COMMENTS:

Reviewer #1 (Remarks to the Author):

The authors have addressed my comments and I now recommend publication.

Kevin Myant

Reviewer #2 (Remarks to the Author):

The authors responded well to my former critiques. By performing additional experiments requested by me and the other reviewers, the paper was substantially improved.

Reviewer #3 (Remarks to the Author):

The authors have addressed most of my concerns, but my major concerns remains somewhat unaddressed. In my prior review, the statement was as follows:

"This is a very interesting study that suggests continuous activation of mTORC1 in crypt base columnar intestinal stem cells drives ageing phenotypes in the intestinal epithelium through activation of a p38-p53 pathway. This paper is definitely a strong candidate for publication, however I have a couple of concerns, starting with the most major, involving the radiation experiments. The suggestion that mTORC1 inhibition using Rapamycin somehow enhances regeneration of the intestinal epithelium, both in young and aged (3.5 and 17.5 month) mice, is in stark contrast with numerous published studies from several groups in different locations (e.g., Ashton GH Dev Cell 2010, Sampson LL et al FASEB J March 2016, Guan Y et al. J. Immunology July 2015, Yousefi M et al. Stem Cell Reports March 2018, etc.).

That the authors find the complete opposite effect observed in these numerous studies from multiple independent groups is a bit disconcerting. In the current study, the authors present data from 6 days after radiation injury, which is unusual since in healthy mice (at least in non-aged healthy mice), the epithelium is fully regenerated after this long, even after much higher doses of injury. Further, the histological evidence presented in the study show only high magnification images of a few crypts for each condition.
Ultimately, for the rest of this study to be taken at face value, the authors should reconcile this obvious discrepancy, at the very least by showing low magnification images encompassing many crypt-villus axes, ideally stained for a proliferative marker or BrdU-pulsed, at more relevant time points- 48, 72 hours post-irradiation"

The authors respond:

"We agree that this is a very important issue. It is well established that mTOR signaling is required for cell proliferation during growth and regeneration of various tissues including the intestine villi and ISCs (we had cited those papers in the original submission). Indeed, we found that in Lgr5-CreERT;mTorf/f mice, TAM administration led to quick decrease in villus size and TA cell proliferation (data not shown). Following the suggestions of the reviewer, we have repeated the experiments and harvested the samples 48, 72 hours post-irradiation (5 Gy). We found that mTOR hyperactivation (by Tsc1 deletion or during to aging) rendered ISC/TA cells increased sensitivity to IR-induced decreases in the numbers of crypts and cell proliferation and increase in apoptosis at day 2 or 3 post IR (see new data for 17.5-month-old mice, Tsc1 deficient mice, Tsc1 and Makp14 double KO mice at day 2 or 3 post IR). The more severe damage may directly cause regeneration problems. Moreover,
We found that RAP was able to rescue the increased sensitivity to IR including decreases in the numbers of crypts and cell proliferation and increase in apoptosis at day 2 or 3 post IR. This will certainly help the regeneration process.
We included all these new data (low magnification images) and clarified this issue in the revised manuscript. See new Fig. 1c, 2d, S2c, S5d, S9b"

So I commend the authors for doing these experiments, yet the new data reveal that the central concern remains: young mice treated with rapamycin then irradiated show no difference in the number of regenerative crypt foci in new Figure 1c, and old mice actually show an increase. Again, this is in stark contrast (at least with respect to the young mice) to the current literature (some cited in my original comment), that Rap treatment has been shown by numerous labs to be a potent inhibitor post-IR crypt regeneration. The authors didn't address this discrepancy between their findings, and the opposing findings from these other labs in the literature.

I still think that this stark discrepancy needs to be addressed. What could account for this study observing results opposite to what's in the literature? How do the doses of Rap and radiation and/or timing of treatment and analysis compare between their study and those in the literature? How could one possibly account for this discrepancy? Reviewer #3 (Remarks to the Author):
Thank you for re-evaluating our manuscript. I believe that in the revision we have addressed the concern why mTOR hyperactivation causes regeneration defects in ISCs and villi. That is because mTOR activation increases the sensitivity to IR-induced cell cycle arrest and apoptosis in ISCs, which slow down the regeneration process. Now we provide an answer to the referee’s concern regarding why RAP rescued regeneration defects in old mice but did not affect regeneration in young mice whereas previous studies showed that RAP inhibited villus regeneration.

It is well established that mTOR has both pro-proliferation and pro-aging effects. mTOR inhibitor rapamycin (RAP) also has dual effects; it acutely inhibits growth and regeneration at high concentrations (>5 mg/kg. see Nature. 2012 June 28; 486(7404):

490–495; eLife 2016;5:e16351), yet long term use at low doses can extend the lifespan in several animal models.

The aim of our manuscript is to test the effects of mTOR activation or inhibition on aging of ISCs and intestinal villi rather than its acute effects on ISC growth or regeneration. So we administrated RAP to mice for 1.5 months. We used RAP at concentration of 3 mg/kg body weight (From Sellleck, dissolved in ethanol at final 50 mg/ml as storage solution, diluted to 0.5 mg/ml with H_2O containing 0.25% PEG400 and 0.25% Twin 80 before use) based on our preliminary studies, to treat mice daily via intraperitoneal injection. RAP treatment at this concentration for 1.5 months did inhibit mTOR activation but did not significantly affect the body weight of adult mice including 16-17.5 month-old mice.

Moreover, after completing the 1.5-month-treatment, the mice are rested for 3 more days (no RAP) before doing nutrient absorption, regeneration and other experiments, to eliminate any possible acute effects of RAP as the small molecule agent is known to inhibit a variety of metabolic processes. This suits our goal of testing whether long term RAP treatment can recover the numbers of health ISCs in old mice. Similar experiments were done to young mice, which were used as the control. The loss of RAP in the bloodstream (destruction and/or secretion) after the resting period may explain why not much effect was observed in young mice (Fig. 1c). Yet it rescued the regeneration defects in old mice since the numbers of ISCs in old mice were recovered after long-term RAP treatment.

I still think that this stark discrepancy needs to be addressed. What could account for this study observing results opposite to what's in the literature?
This can be explained by the difference in the experimental designs. While we were looking at the effects of long-term RAP on ISC and villus aging, other studies were looking at the acute effects on ISC proliferation and regeneration.

How do the doses of Rap and radiation and/or timing of treatment and analysis compare between their study and those in the literature? How could one possibly account for this discrepancy?
We used low dose of IR (6 Gy) since it was sufficient to cause obvious and repairable damage in old mice while many other studies used >10 Gy. We chose a medium RAP concentration, which does not cause severe side effects in our settings, while many studies used >5 mg/kg RAP. We checked multiple time points post IR, this is similar to other studies. We believe that the low concentration of RAP and the resting period (without RAP) before experiments produced the discrepancy.

I hope that this information can satisfy the referee. We can add a schematic to show the experimental design for Fig. 1c or describe the protocol in the text. We are sorry for missing this detail in the Methods in the revised manuscript.
